# Autophagy is induced and supports virus replication in Enterovirus A71-infected human primary neuronal cells

**DOI:** 10.1038/s41598-020-71970-3

**Published:** 2020-09-17

**Authors:** Jhao-Yin Lin, Hsing-I Huang

**Affiliations:** 1 Research Center for Emerging Viral Infections, College of Medicine, Chang Gung University, Kwei-Shan, Tao-Yuan, Taiwan; 2 Department of Medical Biotechnology and Laboratory Science, College of Medicine, Chang Gung University, Kwei-Shan, Tao-Yuan, Taiwan; 3 Department of Pediatrics, Chang Gung Memorial Hospital, Linkou, Taiwan

**Keywords:** Microbiology, Neuroscience, Pathogenesis

## Abstract

Enterovirus A71 (EV-A71), which belongs to the family *Picornaviridae,* can invade the central nervous system (CNS) and cause severe CNS complications or death. The EV-A71 antigen has been detected in the neurons in the brains of humans who died from EV-A71 infection. However, the effect of EV-A71 infection on human neuronal cells remains poorly understood. Human neural stem cells (NSCs) and IMR-32 neuroblastoma cells were differentiated into neuronal cells for this study. Although the neuronal cells were permissive to EV-A71 infection, EV-A71 infection did not induce an obvious cytopathic effect on the neuronal cells. EV-A71 infection did not induce apoptosis in neuronal cells. However, autophagy and autophagic flux were induced in EV-A71-infected neuronal cells. The production of autophagosomes was shown to be important for EV-A71 viral RNA (vRNA) replication in neuronal cells.

## Introduction

Enterovirus A71 (EV-A71) was first isolated in California in 1969, and the strain was named BrCr^1^. A large outbreak of EV-A71 infection was reported in Taiwan in 1998 in which more than 120,000 people were infected and 78 children died^2^. EV-A71 outbreaks have been reported in not only Taiwan and China but also Singapore, southern Vietnam, Brunei and Korea^3^. EV-A71 belongs to the *Picornaviridae* family and the *Enterovirus* genus, which includes four human enterovirus species (*Enterovirus* A, B, C and D), three human rhinovirus species (*Rhinovirus* A, B and C) and five other enterovirus species; EV-A71 is a strain of *Enterovirus* A^4^. EV-A71 infection usually causes the common cold or hand, foot, and mouth disease (HFMD) but can also cause severe central nervous system (CNS) diseases, especially in infants and young children under 5 years of age. EV-A71 is a highly neurotropic virus that causes CNS complications, such as encephalitis, aseptic meningitis, brain stem encephalitis, motor neuron death, neurogenic pulmonary edema, hemorrhage, and death^1,5^. In Asia, EV-A71 is an important cause of viral encephalitis, which can affect neurodevelopment and cognitive function. EV-A71-infected patients were reported to have severe CNS complications, including neural sequelae, delayed neurodevelopment, deficient cognitive function, and cardiopulmonary failure after viral infection^6^.

Some neurotrophic viruses infect neuronal cells, causing cell death and inducing an inflammatory response that can be attributed to neuroinflammatory processes in the CNS. Fetal human cases of EV-A71 infection showed severe lesions in the brainstem, spinal cord and thalamus, and the EV-A71 antigen was detected in neurons of the CNS^7–10^. Additionally, the EV-A71 antigen was detected in the spinal cord, cerebellum and motor cortex in EV-A71-infected monkeys^11,12^. However, the effect of EV-A71 infection on neurons is not well understood.

Autophagy is a conserved process by which cellular homeostasis and cellular survival are maintained under stress conditions^13,14^. Autophagy occurs at a basal level under normal cellular conditions but is rapidly upregulated when cells experience stress, such as starvation and pathogen invasion. Long-lived cytoplasmic proteins, dysfunctional organelles or even invasive pathogens are engulfed within double- or multiphospholipid membrane vesicles called autophagosomes and then delivered to lysosomes for degradation^15,16^. The progression of autophagy is divided into several steps: induction, vesicle nucleation, vesicle elongation, autophagosome-vacuole fusion, vesicle breakdown and degradation. The induction step involves mTOR, which inhibits autophagy by phosphorylating Atg13. When mTOR is inhibited, dephosphorylated Atg13 binds to Atg1 and Atg17 to induce autophagy. Lipid-conjugated LC3, named LC3-II, is a major marker of autophagosome formation. Autophagosomes fuse with lysosomes, resulting in autolysosomes. The inner membrane and luminal contents of these autolysosomes are then degraded by lysosomal acid hydrolases, and some amino acids are transported back to the cytosol for protein synthesis and to support normal cell functions^17^. Autophagy is important for the host defense against viral infection^18^. For example, pattern recognition receptors can recognize vesicular stomatitis virus (VSV)^19^ and Rift Valley fever virus (RVFV)^20^, which triggers antiviral autophagy to control viral replication and limit host lethality. However, viruses have evolved some mechanisms to modulate autophagy that result in increased viral survival and replication. Some RNA viruses, such as influenza A virus^21^, dengue virus^22^, poliovirus (PV)^23^, coxsackievirus B3 (CVB3)^24^, hepatitis C virus (HCV)^25^ and Zika virus^26^, have been demonstrated to induce autophagosome formation to promote viral genome replication. EV-A71 infection can induce autophagy in many different cell lines, and the brain tissues of EV-A71-infected mice showed LC3-positive puncta^27,28^.

In this study, we infected neuronal cells derived from human neural stem cells (NSCs) and neuroblastoma IMR-32 cells with EV-A71. We investigated their susceptibility to EV-A71 infection and found that EV-A71 infection induced autophagy but not apoptosis in these neurons. These results show that autophagy plays an important role in EV-A71 replication in neuronal cells.

## Results

### The characteristics of human NSCs and neuroblastoma IMR-32-derived neuronal cells

**Figure 1:**
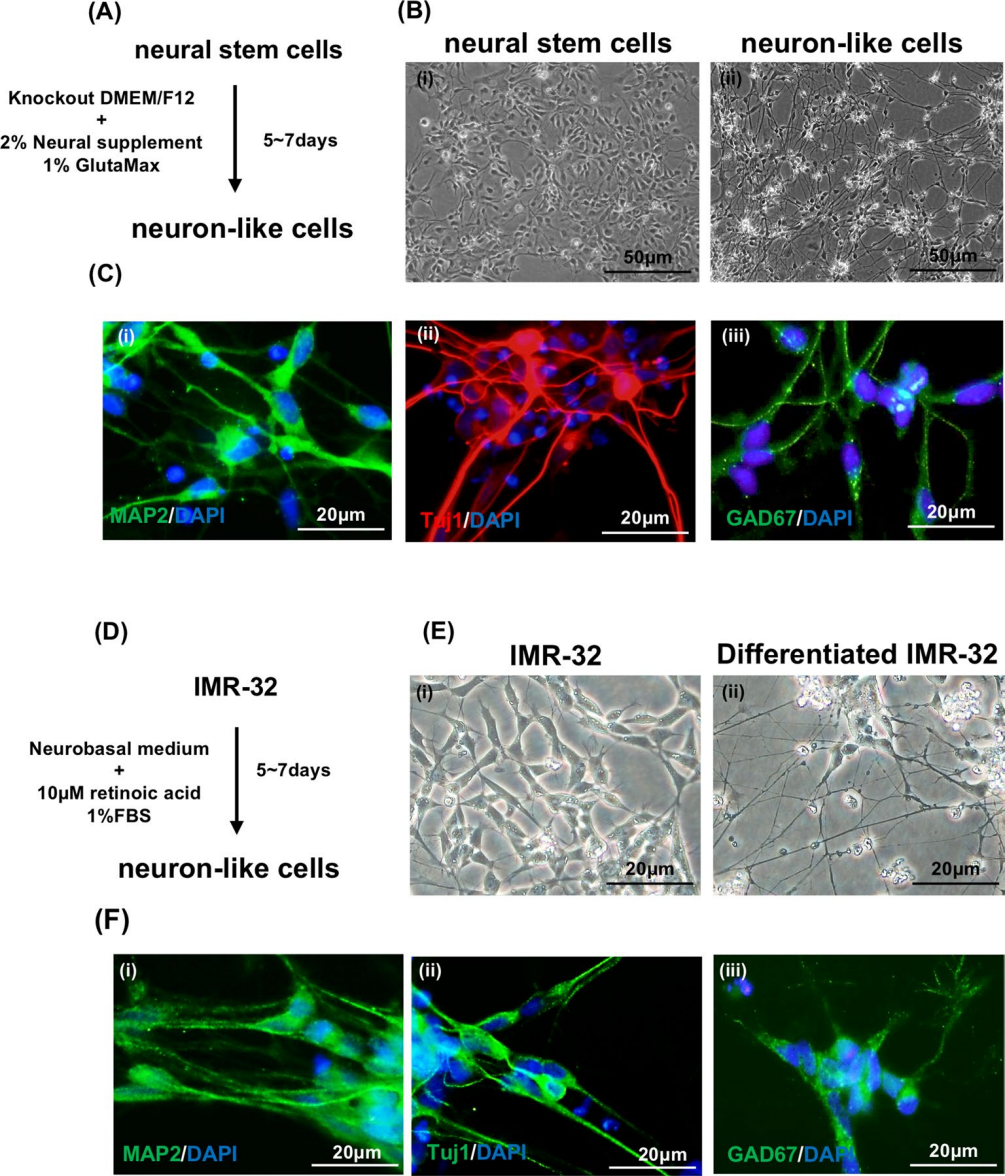
The characteristics of human NSCs and neuroblastoma IMR-32-derived neuronal cells. (**A**) Schematic diagram of neuronal differentiation from human NSCs. (**B**) Bright-field image of human NSCs (i) and neurons (ii), the scale bar represents 50 μm. (**C**) Immunostaining images of neurons derived from human NSCs showing the expression of MAP2 (i), Tuj1 (β-tubulin III) (ii) and GAD67 (iii). The nuclei are counterstained with DAPI. The scale bar represents 20 μm. (**D**) Schematic diagram of neuronal differentiation from neuroblastoma IMR-32 cells. (**E**) Bright-field image of undifferentiated and differentiated IMR-32 cells. (**F**) Immunostaining images of neuronal cells derived from neuroblastoma IMR-32 cells showing the expression of MAP2 (i), Tuj1 (β-tubulin III) (ii) and GAD67 (iii). The nuclei were counterstained with DAPI. The scale bar represents 20 μm.

To understand the effect of EV-A71 infection on neuronal cells, human NSCs were used to perform neuronal differentiation. The human NSCs used in this study were derived from NIH-approved H9 human embryonic stem cells and can differentiate into neurons. The human NSCs were maintained in conditional culture medium without the growth factors EGF and FGF-β for 7 days for neuronal differentiation (Fig. 1A). The neurons formed from human NSC differentiation exhibited long neurites (Fig. 1B). Immunofluorescence assay (IFA) was performed to confirm the differentiation efficiency: neuron-specific class III β-tubulin (Tuj1), which is expressed primarily in the early stage of neuronal differentiation^29^, and microtubule-associated protein 2 (MAP2), which is expressed mainly in neurons, were used as specific markers of neuronal differentiation^30^. Neurons derived from human NSCs expressed MAP2 and Tuj1 (Fig. 1C). In addition, the neurons derived from human NSCs also expressed GAD67, which is a specific marker of GABAergic neurons (Fig. 1C). We also used the other cells to perform neuronal differentiation. The protocol for differentiated neuronal cells from neuroblastoma IMR-32 cells is shown in Fig. 1D, and these neuronal cells also exhibited long neurites (Fig. 1E). The neuronal-specific markers Tuj1 and MAP2 and the GABAergic neuronal marker GAD67 were detected in differentiated IMR-32 cells (Fig. 1F). We also stained neuronal-specific markers in undifferentiated human NSCs and IMR-32 to confirm that these neuronal markers were only expressed in differentiated neuronal cells (Figure S1A andS1B).

### Neuronal cells are permissive to EV-A71 infection

**Figure 2:**
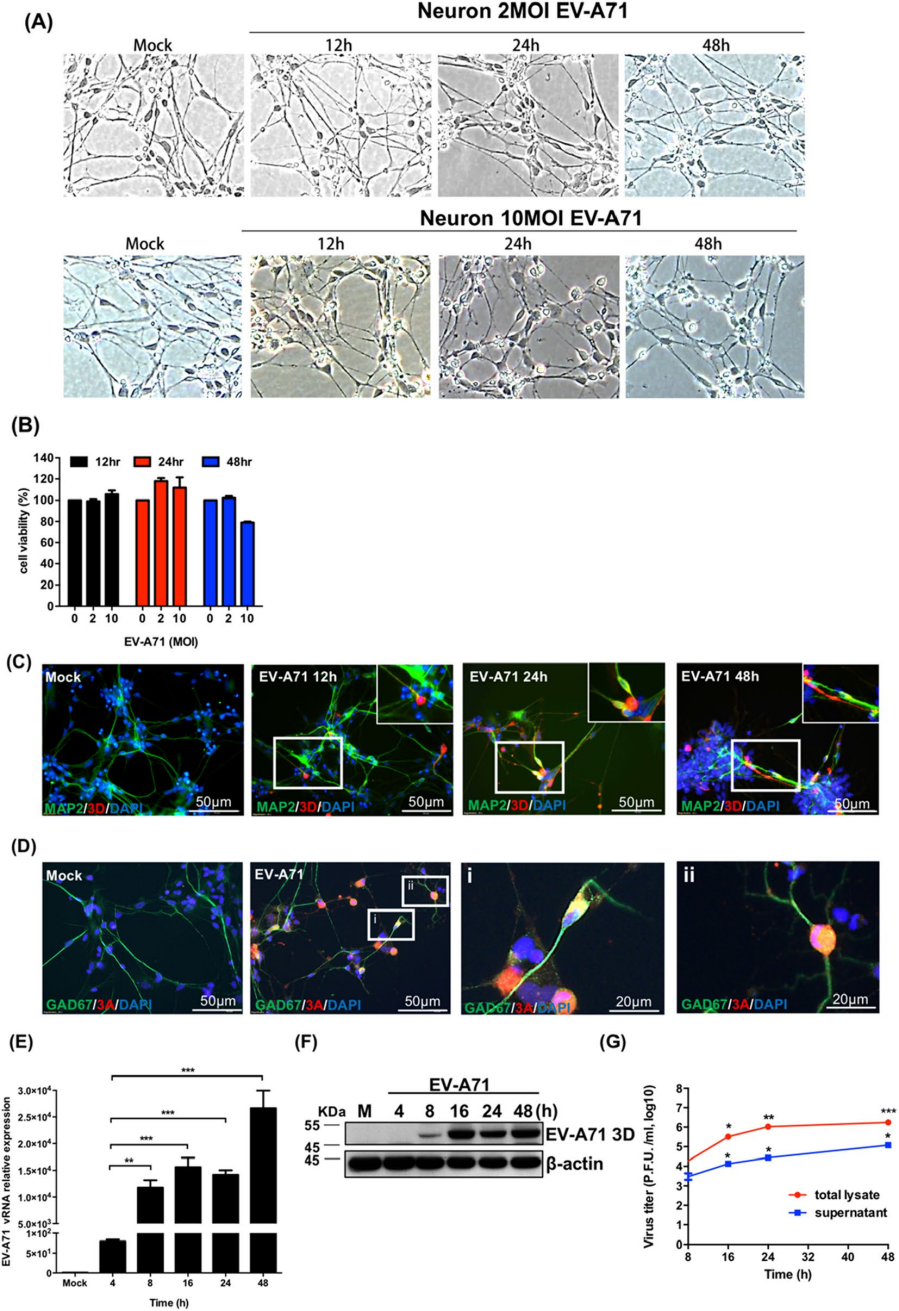
Neuronal cells are permissive to EV-A71 infection. (**A**) Neurons differentiated from human NSCs were infected with EV-A71 at MOIs of 2 and 10, and the cellular morphologies were observed with an inverted microscope (magnification = 200 ×). (**B**) Trypan blue exclusion was performed to quantify the surviving cells at 12, 24 and 48 h postinfection. (**C**) Neurons were infected with EV-A71 at an MOI of 2 or mock infection. Immunofluorescence images of MAP2 and EV-A71 3D ^pol^ in EV-A71-infected neurons at 12, 24 and 48 h postinfection. (**D**) Immunofluorescence images of GAD67 and EV-A71 3A in EV-A71-infected neurons at 24 h postinfection. Cell nuclei were counterstained with DAPI. The scale bar represents 50 μm. Neurons were infected with EV-A71 at an MOI of 2, and total RNA and protein extraction were harvested at 4, 8, 16, 24 and 48 h postinfection. (**E**) RT-qPCR assay was performed to detect the relative levels of EV-A71 5′ UTR vRNA, and (**F**) western blot was used to analyze the expression of EV-A71 3D^pol^ protein. β-actin was used as an internal control. (**G**) The total lysates (supernatant + cells) and supernatant were also harvested to detect the virus titer at 8, 16, 24 and 48 h postinfection with plaque assay. The experiments were performed in triplicate, and the error bars represent the SD. Statistical analyses were performed using Student’s t test. *p < 0.05; **p < 0.01; ***p < 0.001.

To study the susceptibility of human neuronal cells to EV-A71, human NSC-derived neurons were examined for EV-A71 infection. At 12, 24 and 48 h postinfection, the typical cytopathic effect (CPE) of EV-A71 infection was not observed. Few neurons had detached from the culture plates (Fig. 2A). The survival rate of mock- or EV-A71-infected neurons was quantified by a trypan blue assay, which showed no significant difference in cell survival between mock- and EV-A71-infected cells (Fig. 2B). To determine whether EV-A71 could infect neurons, an IFA was performed to evaluate the expression of EV-A71 3D^pol^ in EV-A71-infected neurons. The IFA results revealed the expression of EV-A71 3D^pol^ in MAP2 positive neurons over the course of EV-A71 infection (Fig. 2C). The EV-A71 3A protein was also detected in GAD67-positive neurons (Fig. 2D). EV-A71 replication was also demonstrated by vRNA and viral protein expression and by the viral growth curve. Total RNA was extracted from the neurons infected with EV-A71, and the expression of EV-A71 vRNA was evaluated with RT-qPCR. The relative level of EV-A71 5′ UTR expression increased significantly with infection time (Fig. 2E). Proteins were extracted from EV-A71-infected neurons harvested at various time points, and viral protein expression was measured using western blotting. The expression of the EV-A71 3D^pol^ protein was first detected at 8 h postinfection and was further increased at 16 h postinfection (Fig. 2F). The total lysates and supernatants of the EV-A71-infected neurons were harvested at various time points, and a plaque assay was performed to measure the production of progeny virus. The virus of total lysate (supernatant and cells) increased significantly from 8 to 48 h postinfection. The viral titer of the supernatant also increased with increasing viral infection time (Fig. 2G). The findings indicated that the lack of a CPE was not due to the inability of EV-A71 to enter and replicate in neuronal cells.

### Caspase 3, caspase 8 and caspase 9 are not activated in EV-A71-infected neuronal cells

**Figure 3:**
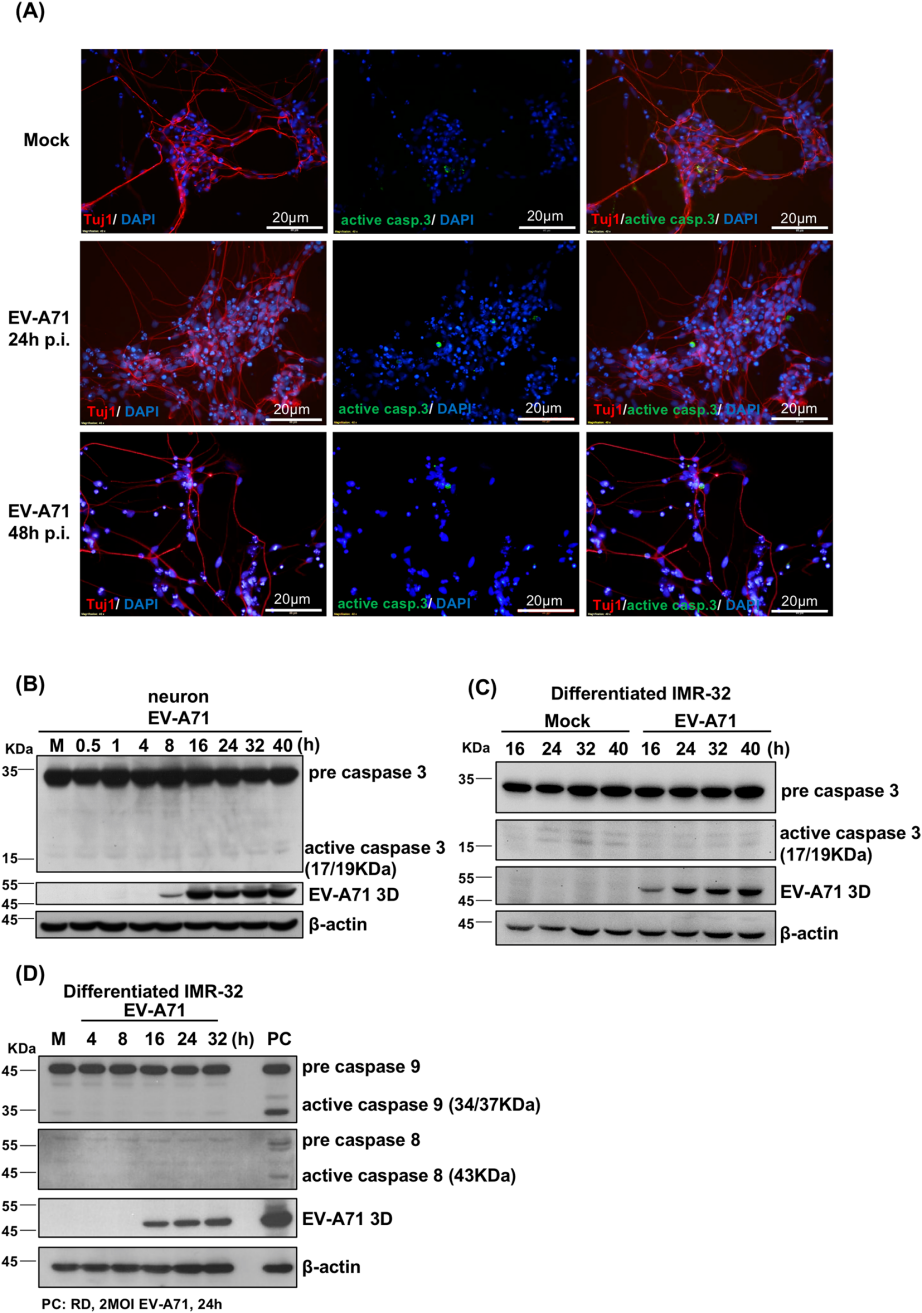
Caspase 3, caspase 8 and caspase 9 are not activated in Enterovirus A71-infected neuronal cells. Neurons derived from human NSCs were infected with EV-A71 at an MOI of 2. (**A**) Immunofluorescence images of active caspase 3 and Tuj1 in EV-A71-infected neurons at 12, 24 and 48 h postinfection. Green indicates active caspase 3, red indicates Tuj1, and DAPI was used to label nuclei. Scale bar represents 20 μm. (**B**) Neurons derived from human NSCs were infected with EV-A71 at an MOI of 2, and total protein was extracted at the indicated time points. Western blotting was performed to measure the expression of precleaved and active caspase 3 and EV-A71 3D^pol^. (**C**, **D**) Neuronal cells derived from IMR-32 cells were infected with EV-A71 at an MOI of 2, and total protein was extracted at various time points. The expression of precleaved and active caspase 3, caspase 9 and caspase 8 was measured with western blotting. β-actin was used as an internal control in all western blots.

Our results revealed that EV-A71 infection did not cause an obvious CPE in neurons derived from human NSCs. To test whether EV-A71 could induce apoptosis in neuronal cells, neurons derived from human NSCs were infected with EV-A71 at an MOI of 2. The neurons were then stained with antibodies against active caspase 3 and Tuj1 at 12, 24 and 48 h postinfection. The IFA results revealed that only a few Tuj1-positive cells also expressed active caspase 3. The expression of active caspase 3 was not increased over the course of EV-A71 infection (Fig. 3A). Western blot assays were performed to measure the expression of the precleaved and active forms of caspase 3. The protein level of active caspase 3 in EV-A71-infected neurons did not increase with infection time (Fig. 3B). Similar results were observed in differentiated IMR-32 cells (Fig. 3C). The expression of active caspase 8 and caspase 9 was also not induced in the EV-A71-infected differentiated IMR-32 cells (Fig. 3D). The inhibition of caspase 3 inhibits EV-A71 replication in rhabdomyosarcoma (RD) cells^31^. In our previous report, treatment with the caspase 3 inhibitor Z-VAD decreased EV-A71 replication in RD cells^32^. However, treatment with the caspase 3 inhibitor Z-VAD did not inhibit viral growth in differentiated IMR-32 cells (Figure S2). EV-A71 infection did not induce apoptosis in the neuronal cells.

### EV-A71 induces autophagosome formation in neuronal cells and differentiated IMR-32 cells

**Figure 4:**
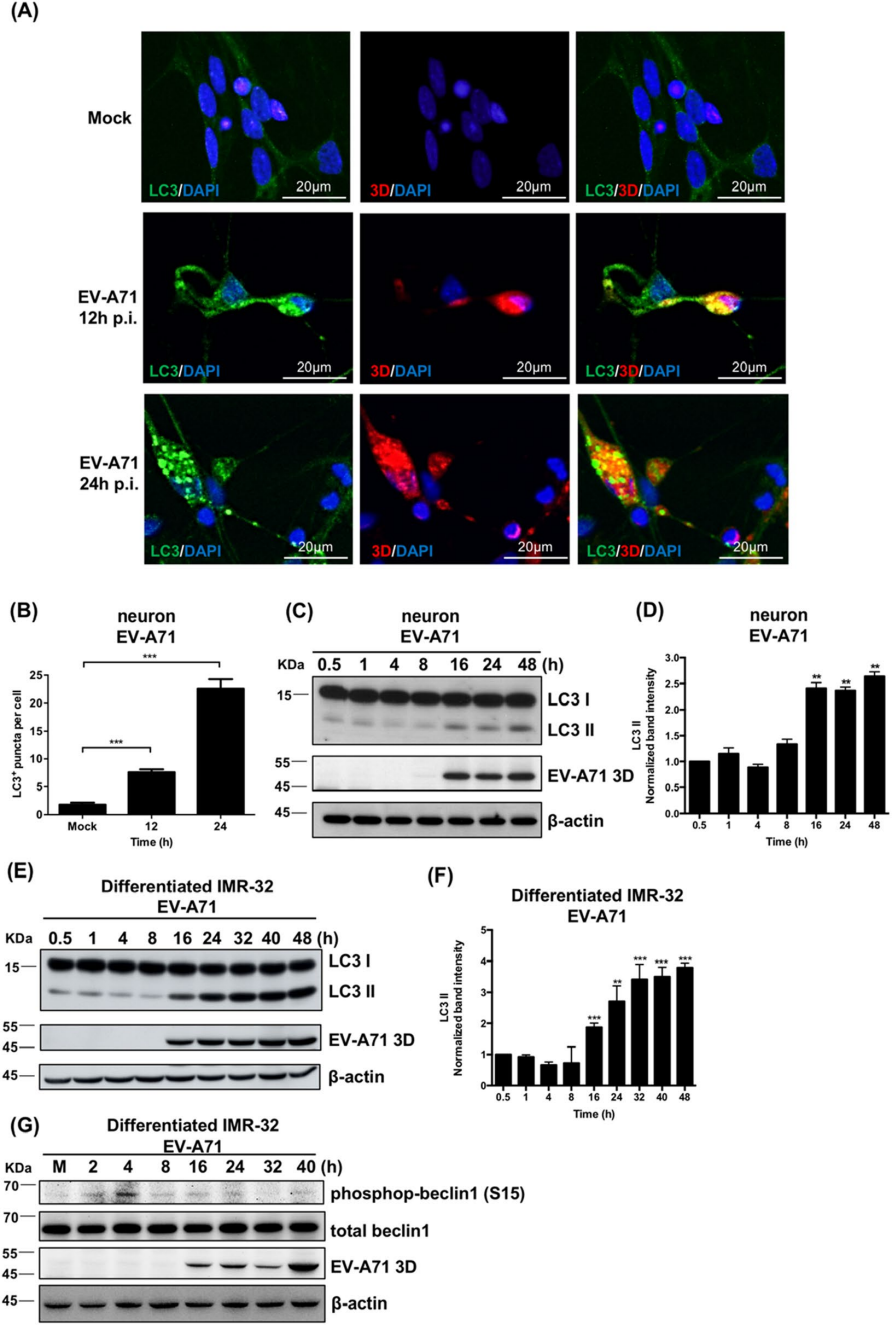
Enterovirus A71 induces autophagosome formation in neuronal cells and differentiated IMR-32 cells. Neuronal cells derived from human NSCs and IMR-32 cells were infected with EV-A71 at an MOI of 2. (**A**) Immunofluorescence images of the LC3 and EV-A71 3D^pol^ in EV-A71-infected neurons at 12 and 24 h postinfection. The green color is LC3, the red color is EV-A71 3D^pol^, and DAPI was used to label nuclei. The scale bar represents 20 μm. (**B**) The average number of LC3^+^ puncta per cell was quantified according to the immunofluorescence results. (**C**) Western blot assays were performed to measure the protein levels of LC3-I, LC3-II and EV-A71 3D^pol^ in EV-A71-infected neurons. (**D**) The band intensities of LC3-II were measured using ImageJ software and normalized to the β-actin. The time point of 0.5 h was used as a reference. (**E**) The protein levels of LC3-I, LC3-II and EV-A71 3D^pol^ of EV-A71-infected differentiated IMR-32 were detected using western blot. (**F**) The band intensities of LC3-II were measured using ImageJ software and normalized to the β-actin. (**G**) The protein levels of phospho-Beclin1 S15, total Beclin1 and EV-A71 3D^pol^ of EV-A71-infected differentiated IMR-32 were detected using western blot. β-actin was used as an internal control in western blots. The error bars represent the SD. Student’s t test. **p < 0.01; ***p < 0.001.

Autophagy is important in the regulation of neuronal physiology^33^. EV-A71 has been reported to induce autophagy in many different cell lines, including neuroblastoma cell lines, but the effect of EV-A71 on autophagy in primary neuronal cells is unclear. Neuronal cells derived from human NSCs were infected with EV-A71, and an IFA was performed to measure the expression of LC3 positive puncta and EV-A71 3D^pol^ at 12 and 24 h postinfection. EV-A71 infection induced the formation of LC3-positive puncta in neurons, and these LC3-positive puncta colocalized with the EV-A71 3D^pol^ protein-expressed cells. (Fig. 4A). The LC3-positive puncta were quantified and significantly increased with increasing infection time in EV-A71-infected neurons (Fig. 4B). During the formation of autophagosomes, LC3-I is conjugated with phosphatidylethanolamine (PE) to form LC3-II, so the level of LC3-II correlates with the number of autophagosomes^34^. Proteins were extracted from EV-A71-infected neurons derived from human NSCs, and the levels of LC3-I and LC3-II were analyzed using western blotting. The protein level of LC3-II significantly increased as infection time increased (Fig. 4C,D). Next, differentiated IMR-32 cells were infected with EV-A71 at an MOI of 2, and proteins were harvested at various time points. Western blot analysis revealed that the protein level of LC3-II was significantly increased with increasing infection time (Fig. 4E,F). The protein level of LC3-II was also increased with increasing viral infection titer (Figure S3A). Different EV-A71 strains also increased the expression level of LC3-II in differentiated IMR-32 cells (Figure S3B). One of the autophagy initiation steps is the activation of the class III phosphatidylinositol 3-kinase (PI3K-III) complex consisting of Beclin1, VSP15, VSP34 and ATG14. To regulate autophagy initiation, Beclin1 must be phosphorylated at several residues, including S15, which is phosphorylated by ULK. Beclin1 S15 phosphorylation is required for PI3K-III complex activation in PtdIns-3P production, which is required for autophagosome formation^35,36^. We measured the expression of phospho-Beclin1 S15 in EV-A71-infected differentiated IMR-32 cells and found that its expression was induced at 4 h postinfection (Fig. 4G). EV-A71 infection induced the formation of LC3 puncta and the protein expression level of LC3-II was increased over the course of EV-A71 infection in the neuronal cells. The results revealed that EV-A71 infection induced autophagy in neurons and differentiated IMR-32, and the initiation of autophagosomes was associated with phospho-Beclin1 S15.

### EV-A71-infected differentiated IMR-32 cells exhibit complete autophagic flux

**Figure 5:**
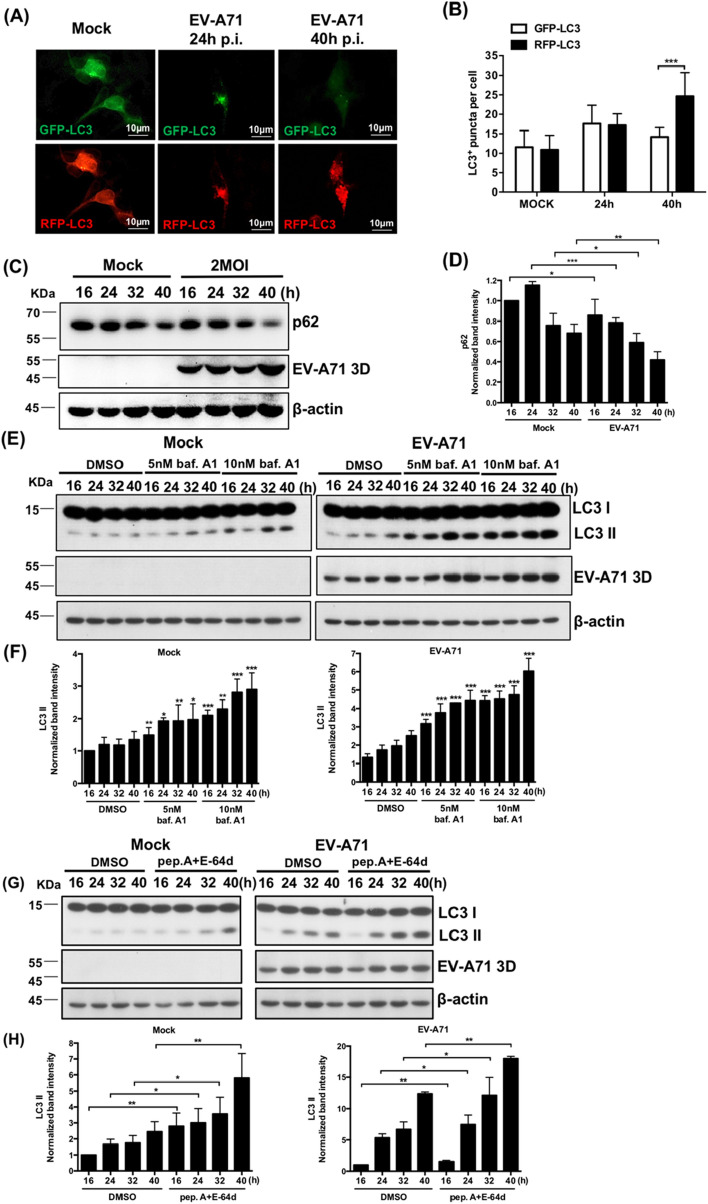
EV-A71-infected differentiated IMR-32 cells exhibit complete autophagic flux. Differentiated IMR-32 cells were infected with EV-A71 at an MOI of 2. (**A**) The autophagy tandem sensor RFP-GFP-LC3B was added to EV-A71-infected differentiated IMR-32 cells and observe the expression of GFP-LC3 and RFP-LC3 at 24 and 40 h postinfeciton. The scale bar = 10 μm. (**B**) The average number of GFP-LC3 puncta and RFP-LC3 puncta was quantified. (**C**) Western blotting was performed to measure the protein levels of p62 and EV-A71 3D^pol^ at indicated time points. (**D**) The band intensities of p62 were measured using ImageJ software and normalized to the β-actin. The time point of mock-infection 16 h was used as a reference. (**E**) Differentiated IMR-32 cells were treated with 5 or 10 nM bafilomycin A1 or with only DMSO and were infected with EV-A71 (MOI = 2) or underwent mock infection. Western blotting was performed to measure the levels of LC3-I, LC3-II and EV-A71 3D^pol^ at indicated time points. (**F**) The band intensities of LC3-II were measured using ImageJ software and normalized to the β-actin. The time point of 16 h was used as a reference. (**G**) Differentiated IMR-32 cells were cotreated with 10 μM pepstatin A and 10 μM E-64d or only DMSO and infected with EV-A71 (MOI = 2) or mock infection. Western blotting was performed to measure the protein levels of LC3-I, LC3-II and EV-A71 3D^pol^ at indicated time points. (**H**) The band intensities of LC3-II were measured using ImageJ software and normalized to the β-actin. β-actin was used as an internal control in all western blots. The error bars represent the SD. Student’s t test. *p < 0.05; **p < 0.01; ***p < 0.001.

Autophagy can be divided into multiple stages, and complete autophagic flux refers to the formation of autolysosomes and their degradation. To test whether autolysosomes are formed in EV-A71-infected differentiated IMR-32 cells, the autophagy tandem sensor RFP-GFP-LC3B was added to EV-A71-infected differentiated IMR-32 cells to monitor autophagy flux. GFP-LC3 is acid sensitive, and RFP-LC3 is acid insensitive. Both GFP-LC3 and RFP-LC3 are visible in autophagosomes. GFP-LC3 is quenched in autolysosomes, which have an acidic pH, but RFP-LC3 is still visible in autolysosomes. RFP expression was higher than GFP expression in EV-A71-infected differentiated IMR-32 cells at 40 h post infection (Fig. 5A). The amount of GRP- and RFP- LC3 puncta was quantified and the RFP-LC3 puncta was significantly more than GFP-LC3 puncta at 40 h post infection (Fig. 5B). These findings indicated that EV-A71 infection induces autolysosome formation in differentiated IMR-32 cells. When the outer membranes of autophagosomes fuse with lysosomes to form autolysosomes, the inner membrane and luminal content of autophagic vacuoles, such as LC3-II, are degraded by lysosomal enzymes^17^. p62 is an ubiquitin-binding scaffold protein that can bind LC3, leading to the degradation of ubiquitinated proteins and its own degradation in autolysosomes^37^. Therefore, a decreased level of p62 is observed in complete autophagic flux. Proteins were extracted from differentiated IMR-32 cells infected with EV-A71 at various time points. Western blot analysis revealed that the level of p62 was significantly decreased in EV-A71-infected cells compared with mock-infected cells over the course of EV-A71 infection (Fig. 5C,D). The expression of p62 was also decreased in the mock-infection. Because neuronal cells have basal autophagy to maintain cellular metabolism and homeostasis, the decreased p62 may be caused by the spontaneous autophagy. In addition, a turnover assay^38^ was performed to investigate the induction of complete autophagic flux. Differentiated IMR-32 cells were treated with bafilomycin A1, which can inhibit the fusion of autophagosomes and lysosomes^39^, and then subjected to EV-A71 or mock infection. Bafilomycin A1 significantly increased the expression of LC3-II in mock- or EV-A71-infected differentiated IMR-32 cells compared with untreated cells (Fig. 5E,F). In addition, differentiated IMR-32 cells were treated with pepstatin A and E-64d, which can inhibit the activity of lysosome protease^40^, and then subjected to EV-A71 or mock infection. Among the mock- and EV-A71-infected differentiated IMR-32 cells, the level of LC3-II was significantly higher in cells treated with pepstatin A and E-64d than in untreated cells (Fig. 5G,H). These results show that EV-A71 infection could induce complete autophagic flux in differentiated IMR-32 cells.

### The induction of autophagy increases EV-A71 vRNA replication in differentiated IMR-32 cells

**Figure 6:**
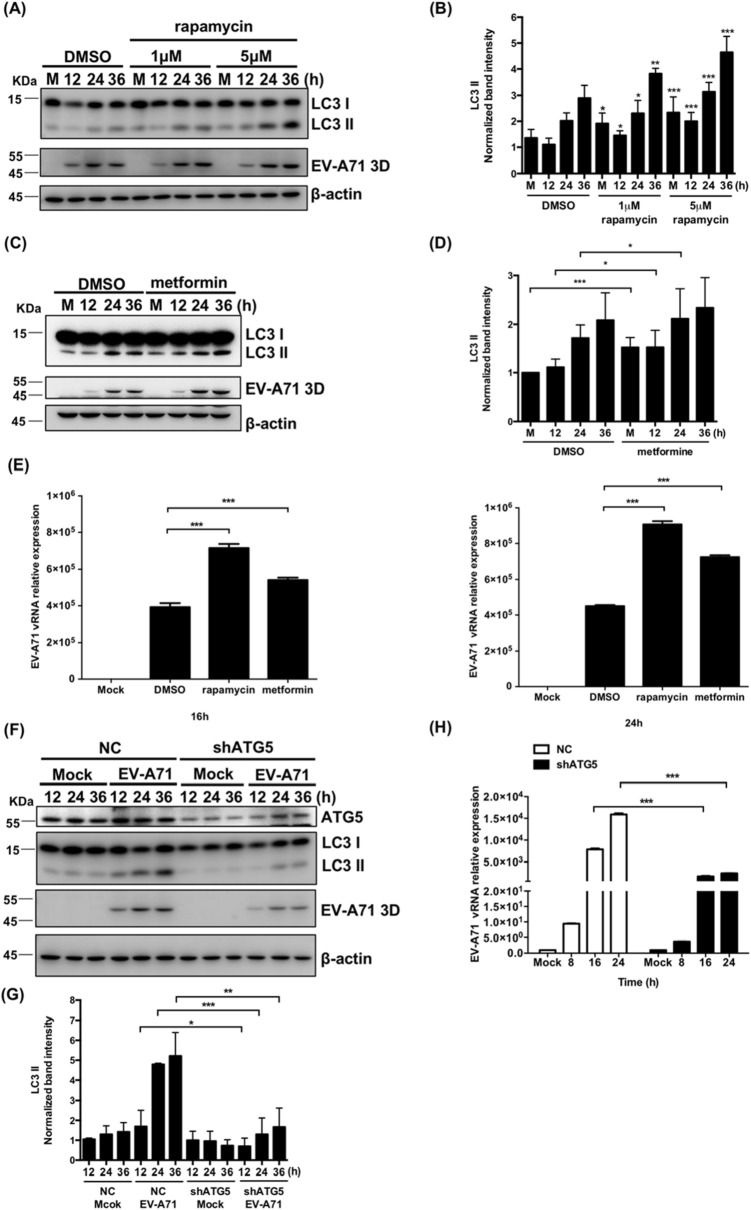
The induction of autophagy increases EV-A71 vRNA replication in differentiated IMR-32 cells. (**A**) Differentiated IMR-32 cells were treated with 1 or 5 μM rapamycin and infected with EV-A71 (MOI = 2) or mock infection. The protein levels of LC3-I, LC3-II and EV-A71 3D^pol^ were measured by western blotting. (**B**) The band intensities of LC3-II were measured using ImageJ software and normalized to the β-actin. The time point of 16 h was used as a reference. (**C**) Differentiated IMR-32 cells were treated with 10 μM metformin and infected with EV-A71 (MOI = 2) or mock infection. The protein levels of LC3-I, LC3-II and EV-A71 3D^pol^ were measured by western blotting. (**D**) The band intensities of LC3-II were measured using ImageJ software and normalized to the β-actin. (**E**) The total RNA was extracted, and RT-qPCR was performed to measure the relative levels of EV-A71 5′ UTR vRNA. (**D**) Differentiated IMR-32 cells transduced with shNC or shATG5 lentivirus were infected with EV-A71 (MOI = 2) or mock infection. Western blot assays were performed to measure the levels of ATG5, LC3-I, LC3-II, and EV-A71 3D^pol^. (**F**) The band intensities of LC3-II were measured using ImageJ software and normalized to the β-actin. (**G**) Total RNA was also extracted for RT-qPCR to measure the relative levels of the EV-A71 5′ UTR. β-actin was used as an internal control in all western blot. The experiments were performed in triplicate, and the error bars represent the SD. Student’s *t* test. *p < 0.05; **p < 0.01; ***p < 0.001.

To determine whether autophagy affects EV-A71 replication in differentiated IMR-32 cells, the cells were treated with two autophagy inducers, rapamycin and metformin, and then infected with EV-A71. Rapamycin induces autophagy by inhibiting the activity of mTOR^41^. The expression level of LC3-II was more significantly upregulated in rapamycin-treated differentiated IMR-32 cells than in DMSO-treated IMR-32 cells over the course of EV-A71 infection (Fig. 6A,B). Metformin, which induces autophagy by activating the AMPK pathway^42^, also increased LC3-II levels in differentiated IMR-32 cells compared with untreated IMR-32 cells during virus infection (Fig. 6C,D). The expression levels of the EV-A71 5′ UTR vRNA were measured with RT-qPCR. The relative EV-A71 5′UTR vRNA levels were significantly upregulated in differentiated IMR-32 cells treated with both autophagy inducers compared with untreated cells (Fig. 6E). To knock down the expression of ATG5 in differentiated IMR-32 cells, small hairpin RNA (shRNA) was delivered by a lentivirus expression system. The cells were transduced with lentivirus expressing shRNA targeting ATG5 or negative control (NC) shRNA, and puromycin was used to select stable clones. Western blotting was performed to confirm the ATG5 knockdown efficiency (Fig. 6F). The expression of LC3-II was also decreased significantly in ATG5-knockdown cells compared to shNC-transfected cells (Fig. 6F,G). This indicated that the formation of autophagosomes was inhibited under this condition. The expression of EV-A71 5′ UTR vRNA was significantly decreased in ATG5-knockdown differentiated IMR-32 cells compared to shNC-transfected differentiated IMR-32 cells (Fig. 6H). This result revealed that the inhibition of autophagy decreased EV-A71 vRNA replication in differentiated IMR-32 cells.

### The accumulation of autophagosomes increases EV-A71 vRNA replication

**Figure 7:**
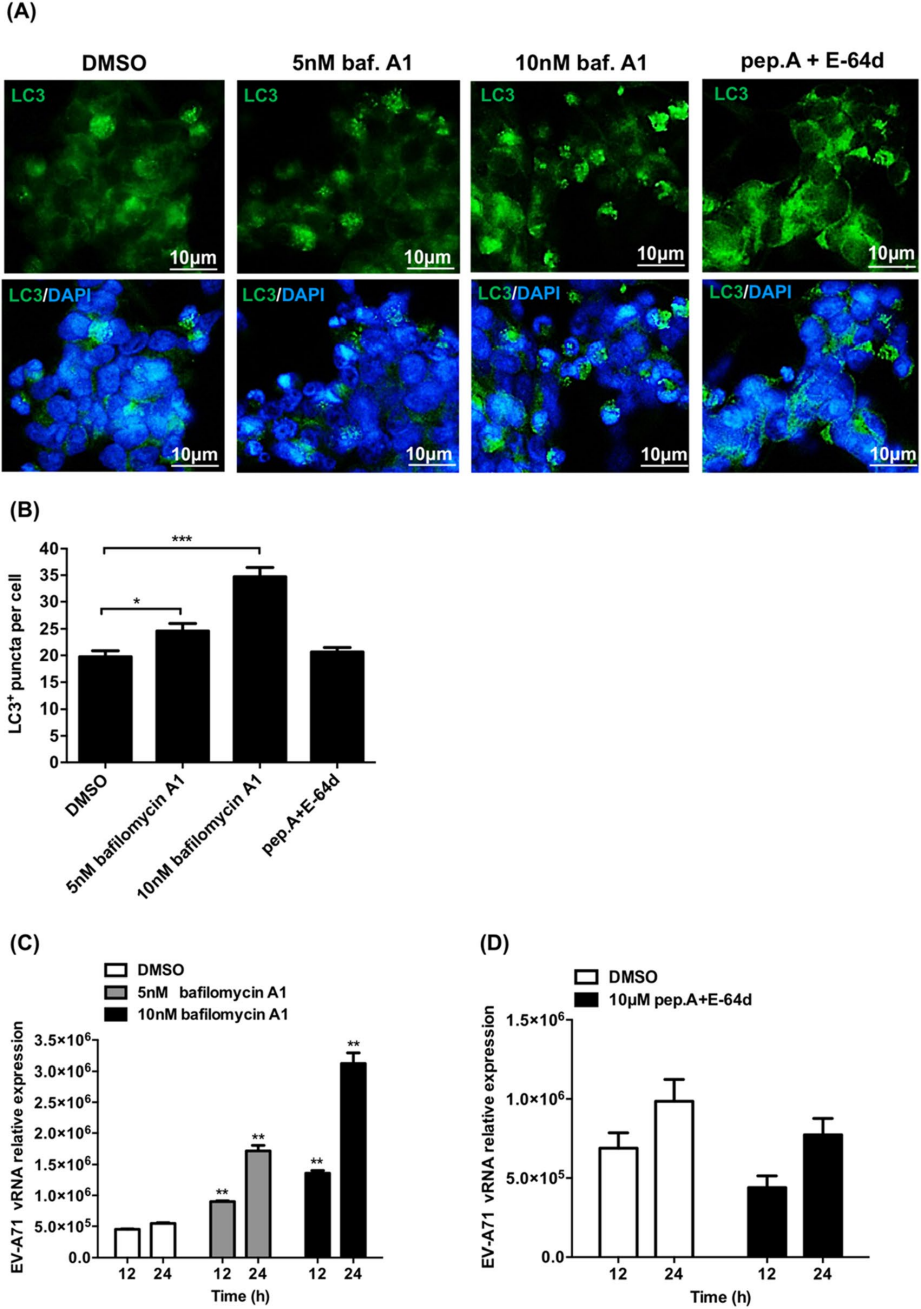
The accumulation of autophagosomes increases Enterovirus A71 vRNA replication. (**A**) Differentiated IMR-32 cells infected with EV-A71 (MOI = 2) and treated with 5 or 10 nM bafilomycin A1 or with 10 μM pepstatin A and E-64d or with DMSO only. Immunofluorescence assay was performed to detect the expression of LC3-positive puncta at 24 h postinfection. Green color is LC3 and DAPI was used to label nuclei. The scale bar represents 10 μm. (**B**) The average number of LC3^+^ puncta per cell was quantified according to the immunofluorescence results. (**C**) Differentiated IMR-32 cells infected with EV-A71 (MOI = 2) or went mock-infection, and treated with 5 or 10 nM bafilomycin A1 or with DMSO only. Total RNA was harvested at 12 and 24 h postinfection, and RT-qPCR assays were performed to measure the relative levels of EV-A71 5′ UTR vRNA. (**D**) Differentiated IMR-32 cells infected with EV-A71 (MOI = 2) or went mock-infection, and treated with 10 μM pepstatin A and E-64d or with DMSO only. Total RNA was harvested at 12 and 24 h postinfection, and RT-qPCR assays were performed to measure the relative levels of EV-A71 5′ UTR vRNA. The experiments were performed in triplicate, and the error bars represent the SD. Student’s *t* test. *p < 0.05; **p < 0.01; ***p < 0.001.

Our results showed that autophagy can support EV-A71 vRNA replication in differentiated IMR-32 cells. To determine which stage of autophagy is important for this phenomenon, differentiated IMR-32 cells were treated with bafilomycin A1 or pepstatin A and E-64d and then infected with EV-A71. We performed the IFA to detect the expression of LC3 positive-puncta in bafilomycin A1-treated or pepstatin A and E-64d- treated differentiated IMR-32 cells infected with EV-A71. Bafilomycin A1 inhibits the fusion of autophagosomes and lysosomes, so the expression levels of LC3-positive puncta were increased significantly in bafilomycin A1-treated cells compared to DMSO-treated controls. The treatment of pepstatin A and E-64d inhibit the protease activity of autolysosomes and prevent the degradation of autolysosome components, but not inhibit the fusion of autophogosomes and lysosomes. Therefore, the expression of LC3- positive puncta was not increased as much as bafilomycin A1 treated- condition (Fig. 7A,B). Total RNA was harvested from cells at various time points, and RT-qPCR was performed to measure the expression of EV-A71 vRNA. The relative amount of EV-A71 vRNA was significantly increased in the bafilomycin A1-treated group compared with the DMSO-treated control (Fig. 7C). In contrast, EV-A71 vRNA was not increased in pepstatin A and E-64d-treated cells (Fig. 7D). Thus, blockade of the fusion of autophagosomes and lysosomes could induce more autophagosome formation and support EV-A71 vRNA replication in differentiated IMR-32 cells.

## Discussion

EV-A71 proteins have been detected in the neuronal cells of EV-A71-infected human brains. However, the effect of EV-A71 infection on human neurons is still unclear. To understand the susceptibility of human neuronal cells to EV-A71 infection, human NSCs were differentiated into neurons for EV-A71 infection. NSCs differentiate into the neuronal cells of the CNS, and this cell model has been used to study the interaction of neuronal cells with neurotropic viruses, such as CVB3^43^, HCMV^44^ and varicella-zoster virus (VZV)^45^. IMR-32 and SH-SY5Y cells can be induced neuronal differentiation through retinoic acid treatment, and differentiated IMR-32 and SH-SY5Y cells express the neuronal markers Tuj1, MAP2 and GAD67, similar to the neurons derived from human NSCs. Differentiated IMR-32 and SH-SY5Y cells have been used to study neurodegenerative diseases, such as Alzheimer’s disease^46^.

The EV-A71 viral proteins 3D and 3A colocalized with the neuronal-specific markers MAP2 and GAD67, which indicated that EV-A71 could infect neurons. EV-A71 vRNA and viral protein expression were detected in EV-A71-infected neurons and viral growth curve was also increased with infection time. These results revealed that EV-A71 could replicate in neurons. However, EV-A71 infection did not induce significant neuronal cell death, and caspase 3 was not activated in neuronal cells derived from human NSCs and IMR-32 cells. Furthermore, caspase 8 and caspase 9 were not activated in EV-A71-infected differentiated IMR-32 cells. These results revealed that EV-A71 infection did not induce caspase-dependent apoptosis in neuronal cells, in contrast to results in diverse EV-A71-infected human cell lines. EV-A71 infection activates the intrinsic apoptosis pathway in neural lineage cells and the extrinsic apoptosis pathway in nonneuronal cells^47^. PV has been indicated to infect primary hippocampal neurons and delay the apoptosis of PV-infected neurons compared to that of nonneuronal cells^48^. VZV can infect neurons derived from human embryonic stem cells and cause persistent nonproductive infection for weeks but does not cause an obvious CPE or apoptosis in virus-infected neurons^45,49,50^. The activation of caspase 3 was shown to promote EV-A71 viral protein translation and viral production in RD cells^31^. However, we found that the inhibition of caspase3 does not decrease EV-A71 growth in differentiated IMR-32 cells. EV-A71 replication in neuronal cells may not be dependent on the activation of caspase 3.

Basal autophagy is important for cellular metabolism and homeostasis, especially in postmitotic cells, such as neuronal cells. Because neuronal cells cannot dilute wastes such as protein aggregates, nonfunctional organelles or organelles damaged by cell division, autophagy is important for neuronal cells to maintain the quality of their proteome and organelles to support their long-term viability^51^. Autophagy also plays an important role in the mechanism by which cells remove invading pathogens. Virus infection, including enterovirus infection, has been shown to induce autophagy^52^. In our study, EV-A71-infected neuronal cells showed more LC3-positive puncta in the cytosol than did mock-infected neuronal cells. LC3-positive puncta are formed in the process of autophagy and indicate the formation of autophagosomes. The number of LC3-positive puncta in neuronal cells increased with infection time. Western blot assays revealed that the expression of LC3-II was increased in neuronal cells over the course of EV-A71 infection.

We found that the expression of LC3-II continuously increased in EV-A71-infected neurons and in differentiated IMR-32 cells. However, the expression of LC3-II was decreased in EV-A71-infected neuroblastoma SK-N-SH cells in a late stage of infection^28^. This increased expression of LC3-II may be due to increased autophagy induction or may indicate the inhibition of autophagosome and lysosome fusion. The autophagy tandem sensor GFP-RFP-LC3B was added to EV-A71-infected differentiated IMR-32 cells to confirm autophagic flux. GFP-LC3 is acid sensitive, and RFP-LC3 is acid insensitive. Both GFP-LC3 and RFP-LC3 are visible in neutral pH autophagosomes. GFP-LC3 is quenched in autolysosomes, which have an acidic pH, but RFP-LC3 is still visible in autolysosomes. RFP-LC3 expression was higher than GFP-LC3 expression in EV-A71-infected differentiated IMR-32 cells at a late stage of infection. That revealed the autolysosomes formation. We used bafilomycin A1 to inhibit the fusion of autophagosomes and lysosomes and used pepstatin A1 and E-64d to inhibit the protease activity of autolysosomes. Both conditions can prevent the degradation of autophagosomal components, such as LC3-II, in autolysosomes. If the autophagic flux is complete, the expression level of LC3-II is increased with drug treatment. In contrast, if the expression level of LC3-II is not changed with drug treatment, complete autophagic flux does not occur. In our results, the expression of LC3-II was significantly increased under both conditions compared to the untreated control condition, indicating that complete autophagic flux occurred in EV-A71-infected neuronal cells. Autophagy can deliver viral components, viral particles or the host factors required for viral replication to lysosomes for degradation and can suppress virus replication^53^. However, viruses have developed molecular strategies to avoid their recognition and degradation via autophagy and can even exploit autophagy for their benefit^54^. CVB3 has been indicated to inhibit the transcription and translation of syntaxin 17 and SNARE to block the fusion of autophagosomes and lysosomes, preventing degradation via autophagy^55^. The CVB3 2A protease can cleave p62 to evade detection and autophagy-mediated degradation. CVA16 infection also causes incomplete autophagic flux in infected cells^56^. PV is detected by the host protein galectin 8, which subsequently initiates autophagy to degrade the viral genome; however, PV can use the host protein HRAS-like suppressor 3 (PLA2G16) to avoid autophagy detection^57^. However, our results indicated that EV-A71 infection did not inhibit the degradative function of autophagy in differentiated IMR-32 cells.

In this study, we found that EV-A71 vRNA replication was increased in differentiated IMR-32 cells treated with autophagy inducers. In contrast, EV-A71 vRNA replication was decreased in differentiated IMR-32 cells in which autophagy was inhibited. RNA viruses can exploit the double-membrane structure of autophagosomes to proceed with their replication because autophagosomes supply a membrane-bound and protective environment^54^. Autophagy also supplies metabolites and energy for virus replication. Autophagy can support EV-A71 replication in human cell lines. There is no direct evidence to indicate which stage of autophagy is involved in this mechanism. Therefore, differentiated IMR-32 cells were treated with bafilomycin-A1 to block the fusion of autophagosomes and lysosomes, after which additional autophagosomes accumulated in the cells. Under this condition, EV-A71 vRNA replication was significantly increased compared with that under the DMSO-treated control condition. The EV-A71 3D^pol^ protein level was also increased in bafilomycin-A1-treated differentiated IMR-32 cells compared with control differentiated IMR-32 cells. In EV-A71-infected RD cells, the blockade of autolysosome fusion by chloroquine and bafilomycin A1 was shown to inhibit viral replication^58^. The fusion of autophagosomes and lysosomes requires v-ATPases to form an acidic environment. The acidic environment in vesicles is critical for the activation of proteases and hydrolases. PVs require vesicular acidification for the cleavage of VP0 to form VP2 and VP4^58^. The acidic environment in vesicles is important for viral replication in EV-A71-infected RD cells. However, vesicular acidification may not be the major mechanism that regulates EV-A71 replication in neuronal cells. Pepstatin A and E-64d were used to inhibit the activity of lysosomal enzymes, but not autolysosomes formation. The LC3 positive puncta expression of pepstatin A and E-64d-treated cell was not as much as bafilomycin A1-treated cells. Under this condition, EV-A71 vRNA replication and the viral protein EV-A71 3D^pol^ were not increased. The results in PV- and EV-A71-infected RD cells were similar^59^ and indicate that autolysosomes cannot degrade the factors required for EV-A71 replication.

In this study, we demonstrate that differentiated neuronal cells are permissive to EV-A71 infection. However, apoptosis was not induced, while autophagy was induced in EV-A71-infected differentiated neuronal cells, which regulated EV-A71 vRNA replication. Blockade of the fusion between autophagosomes and lysosomes increased the expression of EV-A71 vRNA in differentiated neuronal cells. This finding indicates that autophagosome accumulation can increase EV-A71 vRNA replication in differentiated neuronal cells.

## Methods

### NSC culture

The human NSCs used in this study were derived from NIH-approved H9 human embryonic stem cells (Thermo Fisher Scientific, MA, USA). The cells were handled following a kit’s protocol. The complete medium used to maintain the human NSCs consisted of 1 × KnockOut D-MEM/F12, 2 mM GlutaMAX-I supplement, 20 ng/ml FGF-β, 20 ng/ml EGF and 2% StemPro neural supplement (all from Thermo Fisher Scientific, MA, USA). Adherent human NSCs were thawed and subcultured in plates precoated with a matrix consisting of CELLStart substrate and D-PBS containing calcium and magnesium (both from Thermo Fisher Scientific, MA, USA). The cells were incubated at 37 °C with 5% CO_2,_ and the medium was replaced with new complete medium every 2 days. When the NSCs were approximately 90% confluent, they were ready to be passaged. The spent medium was removed from the cells, and the cellular surface was rinsed with 1 × D-PBS. To detach the cells, they were treated with Accutase and incubated at 37 °C in a 5% CO_2_ incubator for 1–2 min, after which complete medium was added to stop the dissociation reaction. The cells were gently pipetted several times, transferred to a 15 ml tube, and centrifuged at 200 × *g* for 5 min. The supernatant was aspirated, and the cells were resuspended in complete medium and seeded in precoated plates for maintenance.

### Cell line culture and viral amplification

IMR-32 neuroblastoma cells were cultured in Dulbecco’s modified Eagle’s medium (DMEM) supplemented with 10% fetal bovine serum (FBS) and 1% L-glutamine (all from Thermo Fisher Scientific, MA, USA). Human RD cells were cultured in DMEM supplemented with 10% FBS, 1% L-glutamine, 1% nonessential amino acids and 1 × penicillin/streptomycin (P/S) (all from Thermo Fisher Scientific, MA, USA). The cells were maintained at 37 °C in a 5% CO_2_ incubator. A clinically isolated strain of EV-A71 (TW/Tainan/4643/98) was amplified in RD cells. The virus titer was detected with a plaque assay.

### Neuronal differentiation assay

For neuronal differentiation, human NSCs were seeded in a CELLStart-coated plate and incubated for 2 days. The culture medium was removed, and knockout DMEM/F12 with 2% StemPro neural supplement and 1% GlutaMAX (all from Thermo Fisher Scientific, MA, USA) was used for neural differentiation. The cells were incubated for 5–7 days at 37 °C and 5% CO_2_. IMR-32 cells were seeded in 12-well plates and incubated for 2 days. The medium was exchanged for neurobasal medium (Thermo Fisher Scientific, MA, USA) supplemented with 10 μM retinoic acid (Sigma-Aldrich, MO, USA), 1% FBS and 1% L-glutamine (Thermo Fisher Scientific, MA, USA), and the cells were incubated for 5–7 days at 37 °C and 5% CO_2_.

### Quantification of cell viability

The number of surviving cells at various time points were counted with a trypan blue exclusion assay (Sigma-Aldrich, MO, USA). Live cells were visualized and counted with a hemocytometer. The cells under each condition were counted three times.

### Reagent treatment

Stock solutions of the autophagy inducers rapamycin (Millipore, MA, USA) and metformin (Sigma-Aldrich, MO, USA) at a concentration of 100 mM were prepared in DMSO or sterilized water. Cells were pretreated with these autophagy inducers for 2 h and then infected with EV-A71 for 1 h. After adsorption, fresh medium containing rapamycin or metformin was added to the cells. A 100 μM bafilomycin A1 (Sigma-Aldrich, MO, USA) solution was prepared in DMSO. The cells were treated with bafilomycin A1 during and after EV-A71 infection. Stock solutions of pepstatin A and E-64d (Sigma-Aldrich, MO, USA) at a concentration of 7.3 mM were prepared in DMSO. Cells were simultaneously treated with both drugs and infected with EV-A71. In addition, the cells were treated with both drugs after infection until the indicated time points.

### Knockdown of Atg5 expression by lentivirus-delivered shRNA

Lentivirus-based shRNA constructs targeting human ATG5 were obtained from the National RNAi Core Facility, Taiwan, and a negative control construct (shNC) was obtained from Dr. Shih Shin-Ru. To prepare the lentivirus, 293 T cells were transfected with pLKO-shRNA and two helper plasmids, pMD. G and pCMVΔ8.91, with Mirus transfection reagent. After 48 h, the supernatant was collected and stored at − 80 °C. To generate a stable ATG5-knockdown IMR-32 clone, lentivirus expressing shATG5 was used to transfect IMR-32 cells, and puromycin (1 μg/ml) was used to select cells expressing shATG5.

### EV-A71 infection

The differentiation media of neuronal cells were removed, and the cells were washed once with PBS. Virus stock was diluted in neuronal differentiation medium and added to the cells for adsorption at 37 °C in a 5% CO_2_ incubator. After 1 h, the viral solution was removed, and the cells were washed with PBS. Fresh culture medium was added to cells, which were incubated at 37 °C in a 5% CO_2_ incubator.

### Transduction of GFP-RFP-LC3

A Premo autophagy tandem sensor RFP-GFP-LC3 kit (Thermo Fisher Scientific, MA, USA) was used to monitor the autophagic flux in EV-A71-infected neuronal cells. A total of 10,000 cells were added with 2 μl of the tandem sensor LC3-FP. The reagent was mixed gently with fresh culture medium and added to cells. The cells were incubated at 37 °C in a 5% CO_2_ incubator overnight (> 16 h). Cells were imaged with an Olympus BX51 fluorescence microscope (Olympus, Tokyo, Japan).

### Immunofluorescence

Human NSCs and differentiated neuronal cells were seeded on chamber slides. The cells were fixed with 4% paraformaldehyde for 15 min and then permeabilized with 0.5% Triton X-100 in TBS for 5 min at room temperature. TBS with 2% FBS was used for blocking. The cells were incubated with the following primary antibodies at 4 °C overnight: rabbit anti-SOX2 (1:200, Cell Signaling, CA, USA), mouse anti-nestin (1:200, Millipore, MA, USA), rabbit anti-MAP2 (1:200, Millipore, MA, USA), mouse anti-Tuj1 (anti-neuron-specific class III β-tubulin, 1:200, Millipore, MA, USA), mouse anti-GAD67 (1:200, Millipore, MA, USA), rabbit anti-active caspase 3 (1:200, Millipore, MA, USA), rabbit anti-LC3A/B (1:200, Cell Signaling, CA, USA), mouse anti-EV-A71 3D (1:500, Genetex, CA, USA) or rabbit anti-EV-A71 3A (1:500). The cells were then washed with TBS and incubated with the following secondary antibodies for 1 h at room temperature: DyLight 488-conjugated goat anti-rabbit secondary antibody or DyLight 594-conjugated donkey anti-mouse secondary antibody (1:1,000, Jackson ImmunoResearch Laboratories, Pennsylvania, USA). The cells were then washed with TBS, and cell nuclei were counterstained with 4′,6-diamidino-2-phenylindole (DAPI) (Sigma-Aldrich, MO, USA). Images were collected with an Olympus BX51 fluorescence microscope (Olympus, Tokyo, Japan) and an LSM 510 microscope (Zeiss, Jena, Germany).

### Western blotting

The cells were lysed with protein lysis buffer (1% NP-40, 50 mM Tris and 150 mM NaCl) with 1 × protease inhibitor cocktail and then incubated on ice for 30 min and centrifuged at 13,000 rpm for 10 min at 4 °C. The protein concentration was measured with the Bradford method (Bio-Rad Laboratories, CA, USA). Protein samples were separated by 12% SDS–polyacrylamide gel electrophoresis. Polyvinylidene fluoride (PVDF) membranes (GE, MA, USA) were used for protein transfer. The protein-containing membranes were blocked with 5% skim milk in Tris-buffered saline Tween-20 (TBST: 20 mM Tris–HCl, pH 7.4, 150 mM NaCl, and 0.1% Tween-20) at room temperature for 1 h. The membranes were then incubated with anti-EV-A71 3D (1:800, Genetex, CA, USA), anti-EV-A71 3C (1:500), anti-EV-A71 VP0 (1:2000, Millipore, MA, USA), anti-caspase 3 (1:1,000, Cell Signaling, CA, USA), anti-caspase 9 (1:1,000, Cell Signaling, CA, USA), anti-caspase8 (1:1,000, Cell Signaling, CA, USA), anti-LC3 A/B (1:1,000, Cell Signaling, CA, USA), anti-Beclin1 (1:1,000, Cell Signaling, CA, USA), anti-phospho-Beclin1 S15 (1:1,000, Cell Signaling, CA, USA), anti-p62 (1:1,000, Sigma-Aldrich, MO, USA), or anti-β-actin (1:20,000, Sigma-Aldrich, MO, USA) antibodies. Subsequently, the membranes were incubated with anti-mouse or anti-rabbit horseradish peroxidase-conjugated secondary antibody (1:5,000, Jackson ImmunoResearch Laboratories, Pennsylvania, USA). Horseradish peroxidase was detected with a chemiluminescence reagent kit (Perkin Elmer, MA, USA), and protein signals were detected with a Chemi imaging system (Bio-Rad, CA, USA).

### RNA extraction and RT-qPCR

Total RNA was collected with a TRI reagent solution (Thermo Fisher Scientific, MA, USA) at various time points. The cells were homogenized in TRI reagent solution and mixed with chloroform. The homogenate was incubated for 5 min at room temperature and centrifuged at 12,000 × *g* for 15 min at 4 °C. The aqueous phase, which contained RNA, was transferred to fresh tubes, and an equal amount of isopropanol was added before incubation at − 80 °C overnight. The mixture was centrifuged at 12,000 × *g* for 10 min at 4 °C, and the supernatant was removed. Then, the RNA pellet was washed with 1 ml of 75% ethanol and centrifuged at 7,000 × *g* for 5 min at 4 °C. The 75% ethanol was removed, and the RNA pellet was air dried at room temperature, dissolved in sterile water and incubated at 55 °C to fully dissolve. This RNA solution was stored at − 80 °C. cDNA synthesis was performed with a RevertAid First Strand cDNA Synthesis Kit (Thermo Fisher Scientific, MA, USA). RNA (1–0.5 μg) was used for cDNA synthesis, and the first step was treatment with DNase I at 37 °C for 30 min to remove genomic DNA. EDTA (50 mM) was added to the mixture to inactivate DNase I at 65 °C for 10 min. A random hexamer primer was used for cDNA synthesis, and the protocol was as follows: 65 °C, 5 min; 4 °C, 2 min; 25 °C, 5 min; 42 °C, 80 min; 70 °C, 5 min; and incubation at 4 °C. One microliter of cDNA sample was subjected to qPCR with 5 μM primers, and SYBR green was used to quantify expression. qPCR was carried out in a 384-well plate, and the results were analyzed with a Roche LightCycler 480 system. Each sample was assayed in triplicate, and 18S rRNA was used as a reference gene. We analyzed the relative expression level of each gene by the 2^-∆∆CT^ method. The following primers were used in this study: EV-A71 5′ untranslated region (UTR), forward (5′ CCC TGA ATG CGG CTA ATC C 3′) and reverse (5′ ATT GTC ACC ATA AGC AGC CA 3′) and 18S rRNA, forward (5′ GTA ACC CGT TGA ACC CCA TT 3′) and reverse (5′ CCA TCC AAT CGG TAG TAG CG 3′).

## Supplementary information

Supplementary Information 1.

Supplementary Information 2.

## Data Availability

The data that support the findings of this study are available from the corresponding author upon reasonable request.
