# Supplementary material for: Autophagy is induced and supports virus replication in Enterovirus A71-infected human primary neuronal cells
Source: Sci Rep. 2020 Sep 17;10:15234. doi: 10.1038/s41598-020-71970-3 (PMC7499237; doi:10.1038/s41598-020-71970-3)
Supplement: Supplementary file 1 — Supplementary Information 1. [file 41598_2020_71970_MOESM1_ESM.docx]

**
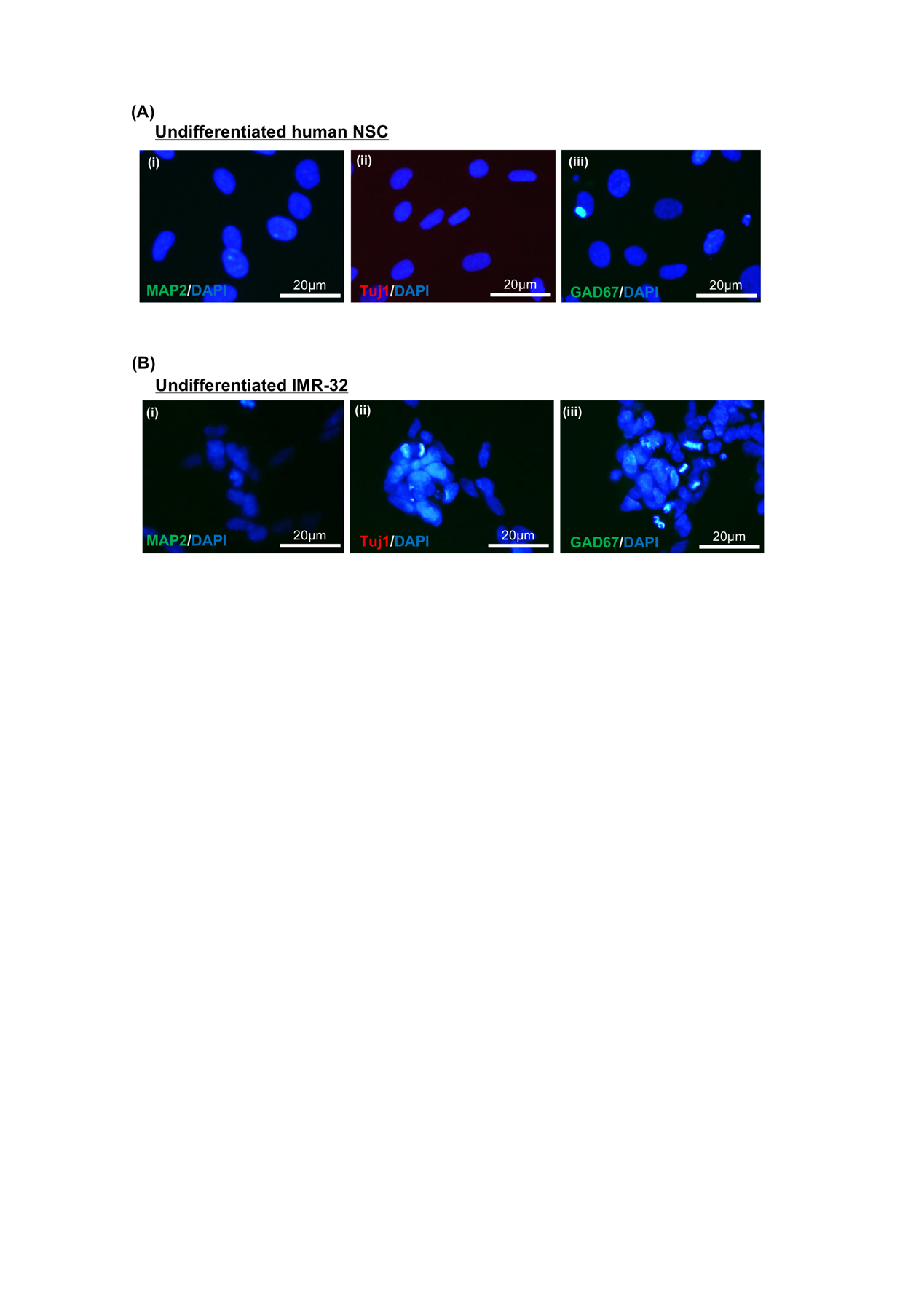
**

**Figure S1. Undifferentiated human NSCs and neuroblastoma IMR-32 cells do not express neuron-specific markers.**

Immunostaining images of human NSCs (A) and IMR-32 cells (B) showing the expression of MAP2 (i), Tuj1 (β-tubulin III) (ii) and GDA67 (iii). The nuclei are counterstained with DAPI. The scale bar represents 20 μm.


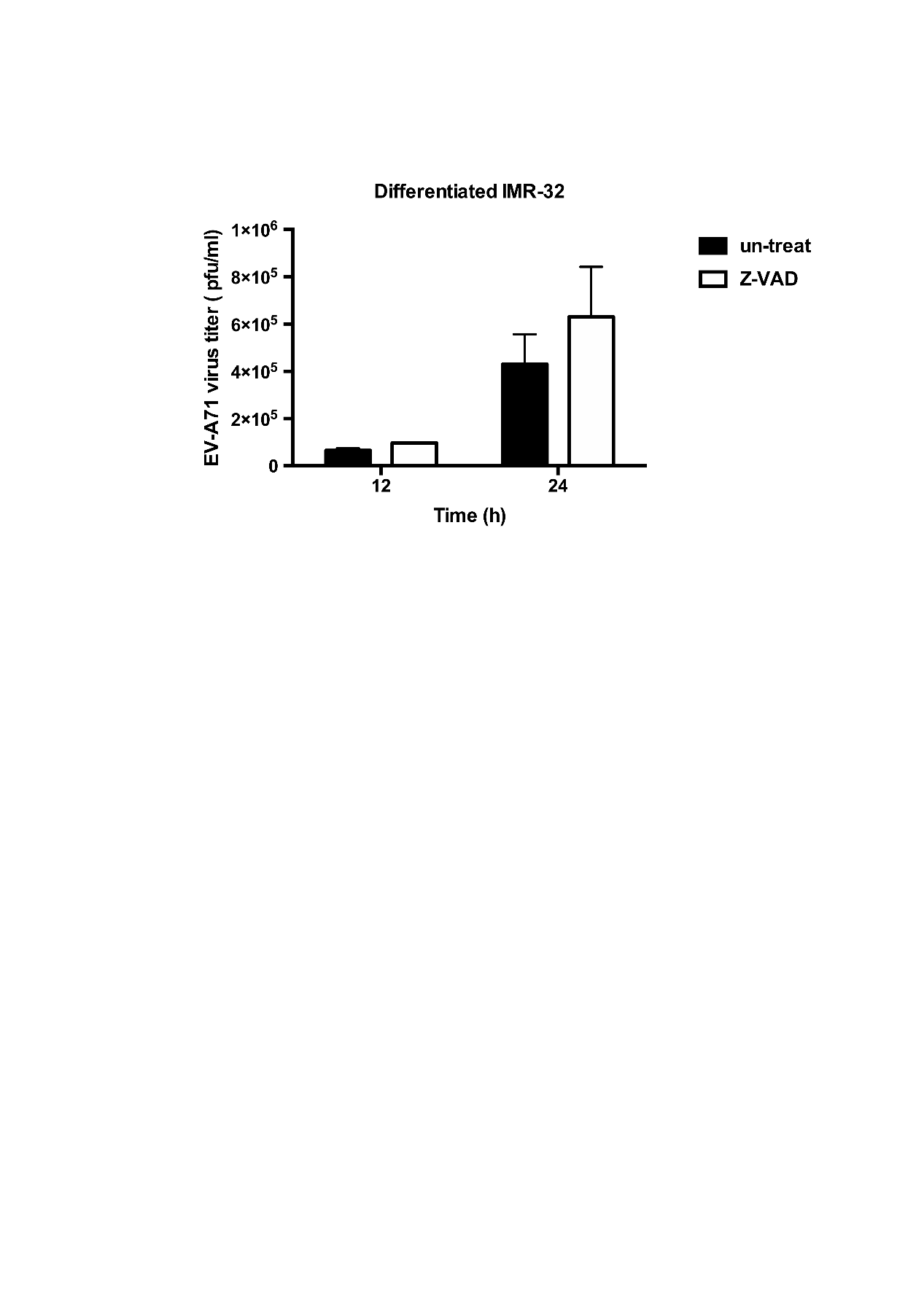


**Figure S2. The inhibition of caspase 3 activity does not attenuate EV-A71 growth in differentiated IMR-32 cells**

Differentiated IMR-32 cells were pretreated with 20 μM Z-VAD for one hour and then infected with EV-A71 at an MOI of 2. Cells were continuously treated with the drug after virus adsorption. The supernatant was harvested at 12 and 24 h postinfection, and a plaque assay was performed to detect the viral titer. The experiments were performed in triplicate, and the error bars represent the SD.


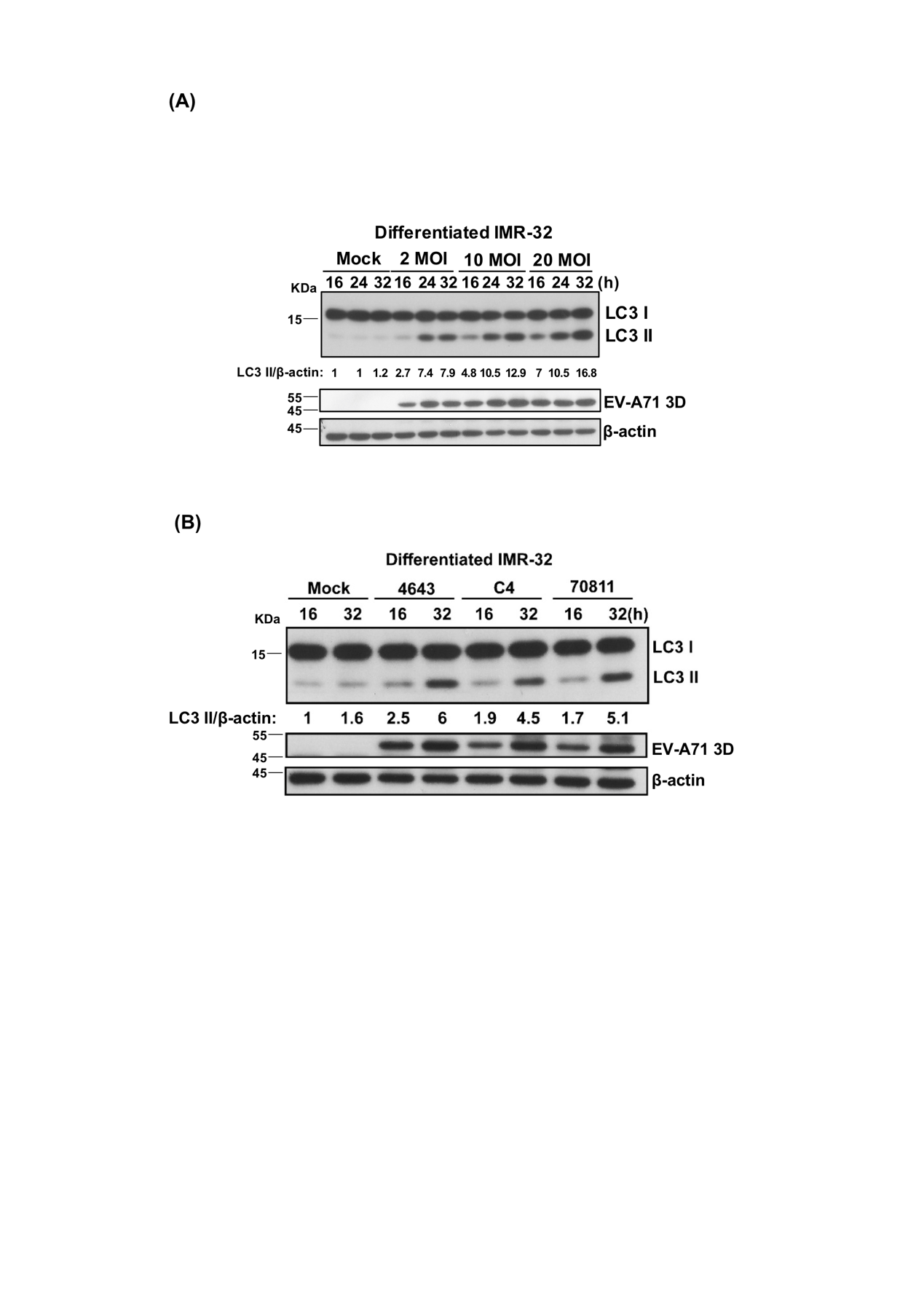


**Figure S3. EV-A71 can induce autophagy in differentiated IMR-32 cells**

(A) Differentiated IMR-32 were infected with EV-A71 at MOIs of 2, 10 and 20. Western blot assay was performed to detected the protein levels of LC3-I, LC3-II and EV-A71 3D^pol^. (B) Differentiated IMR-32 cells were infected with different EV-A71 strains at an MOI of 2. The protein lysates were harvested at 16 and 32 h postinfection. Western blotting was performed to measure the protein levels of LC3-I, LC3-II and EV-A71 3D^pol^. β-actin was used as an internal control.
