# Supplementary material for: Autophagy is induced and supports virus replication in Enterovirus A71-infected human primary neuronal cells
Source: Sci Rep. 2020 Sep 17;10:15234. doi: 10.1038/s41598-020-71970-3 (PMC7499237; doi:10.1038/s41598-020-71970-3)

Autophagy is induced and supports virus replication in Enterovirus A71-infected human primary neuronal cells

Jhao-Yin Lin[1,2] and Hsing-I Huang[1,2,3]

[1] Research Center for Emerging Viral Infections, College of Medicine, Chang Gung University, Kwei-Shan, Tao-Yuan, Taiwan

[2] Department of Medical Biotechnology and Laboratory Science, College of Medicine, Chang Gung University, Kwei-Shan, Tao-Yuan, Taiwan

[3] Department of Pediatrics, Chang Gung Memorial Hospital, Linkou, Taiwan

*Corresponding author: Dr. Hsing-I Huang E-mail: hihuang@mail.cgu.edu.tw

Figure 2F

EV-A71 3D, actin

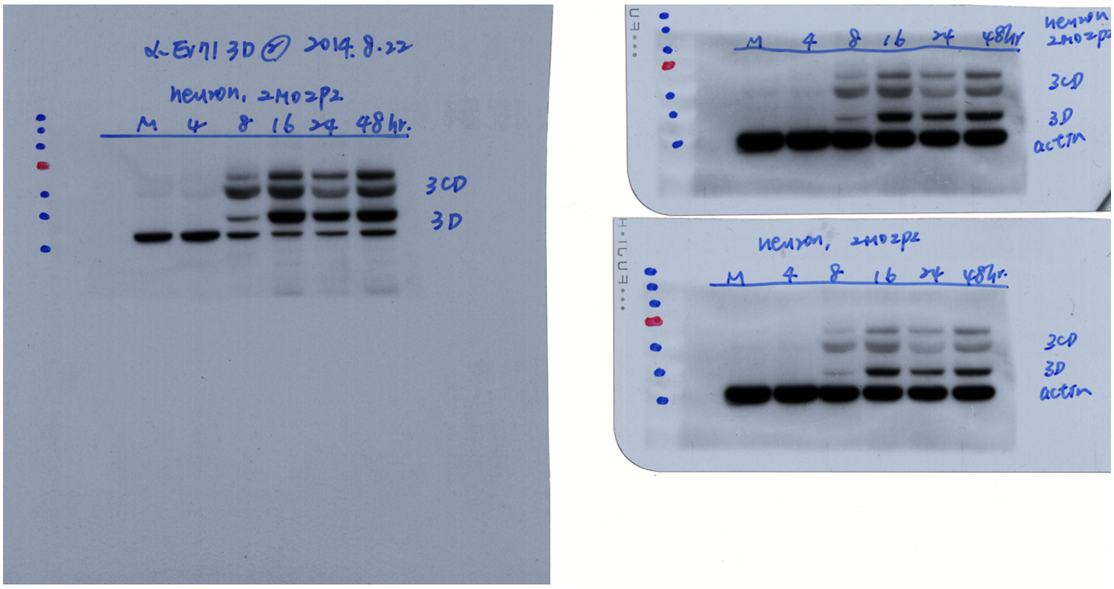

Figure 3B

caspase 3

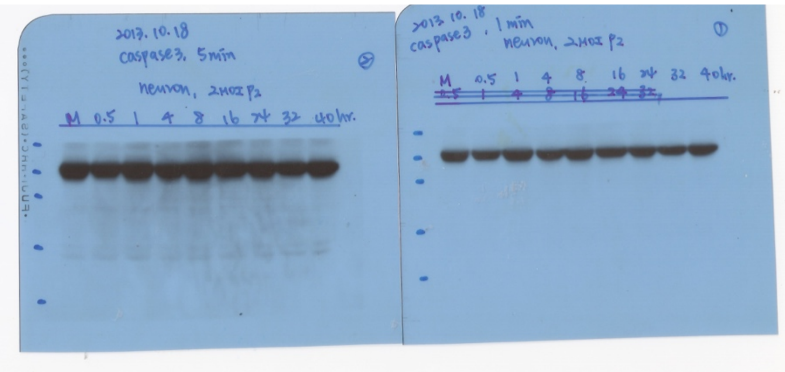

EV-A71 3D, actin

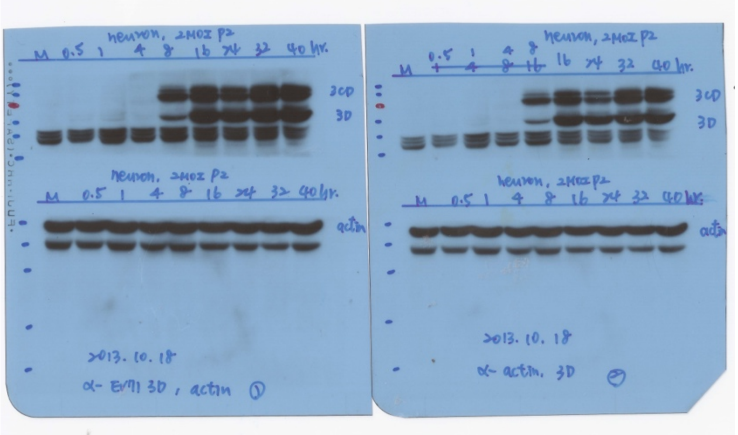

Figure 3C

caspase 3

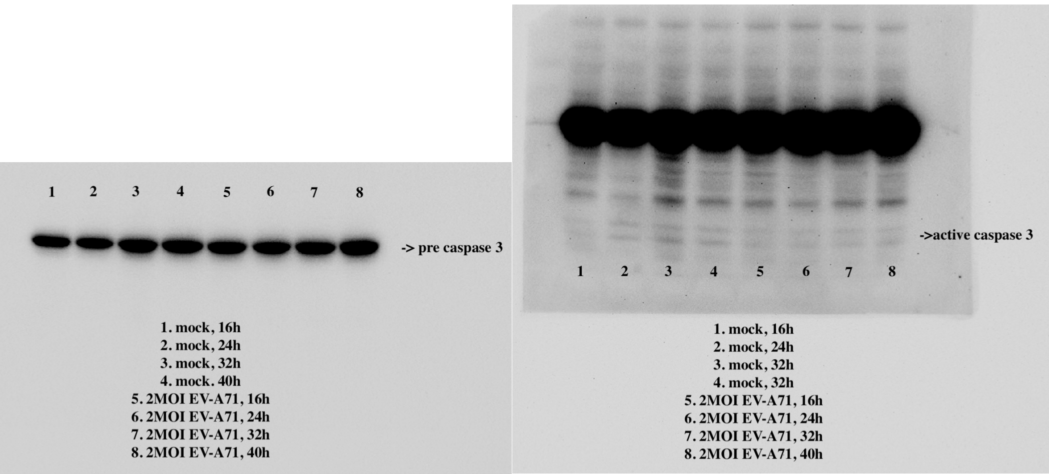

EV-A71 3D

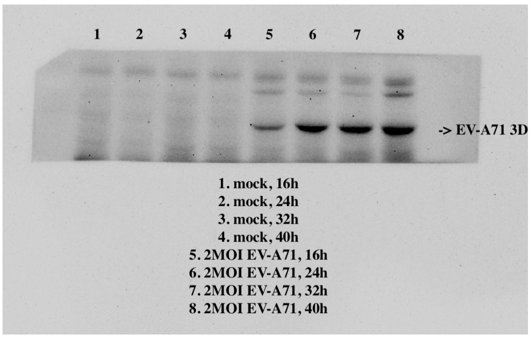

actin

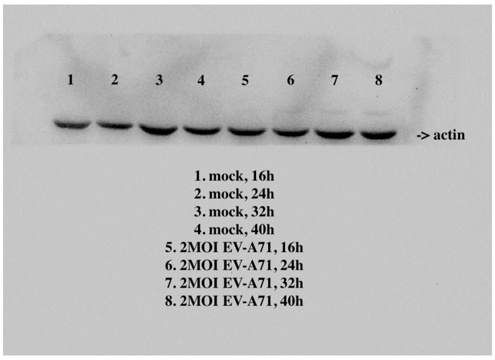

Figure 3D

caspase 9, caspase 8

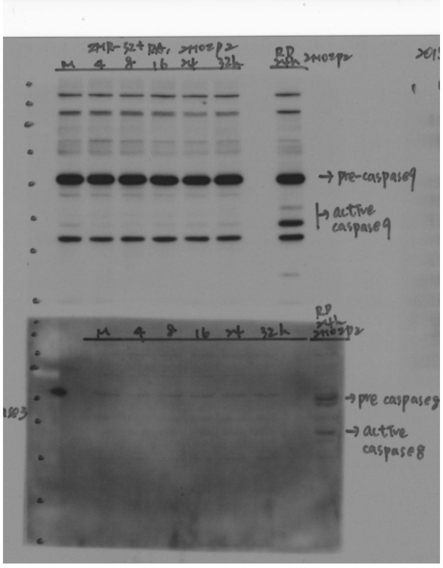

EV-A71 3D

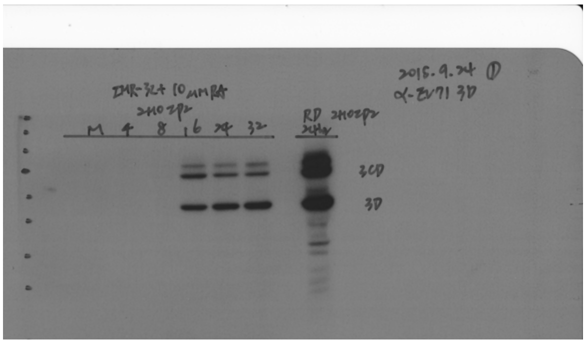

actin

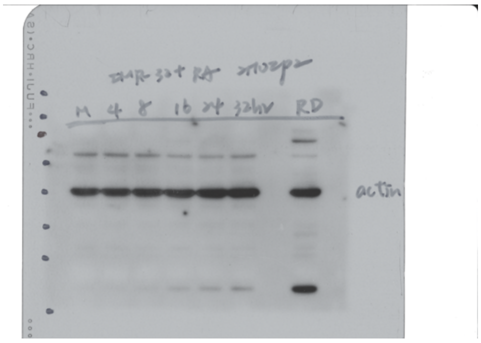

Figure 4C

LC3

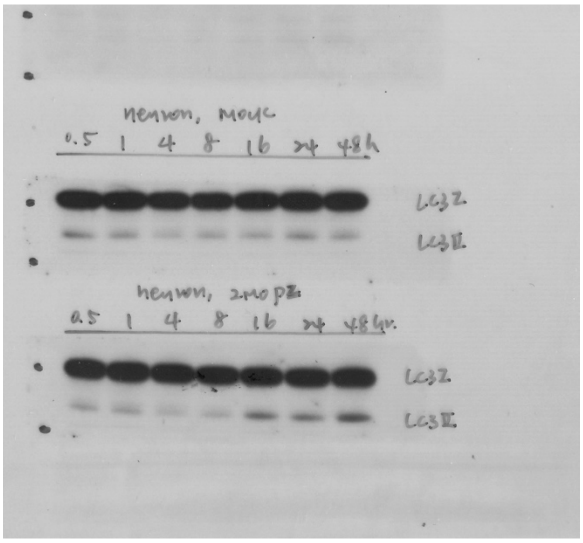

EV-A71 3D

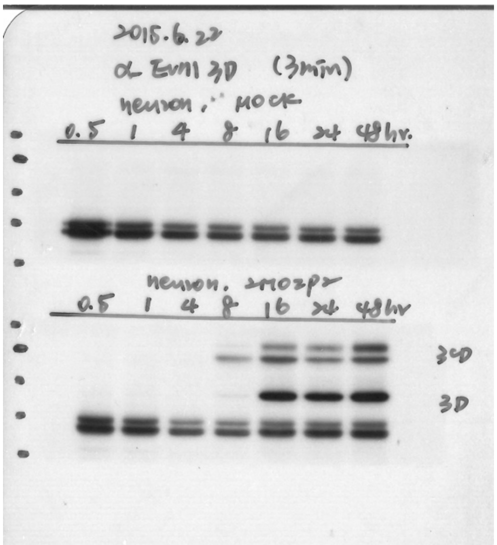

actin

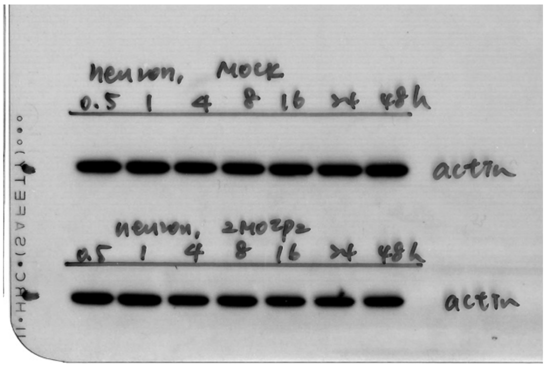

Figure 4E

LC3 I/ LC3II

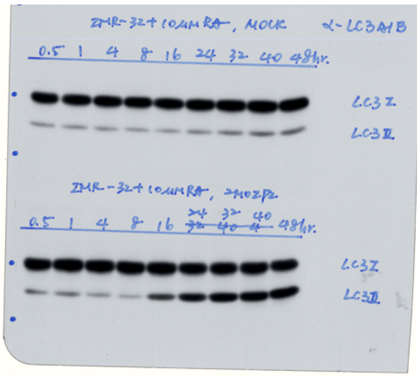

EV-A71 3D, actin

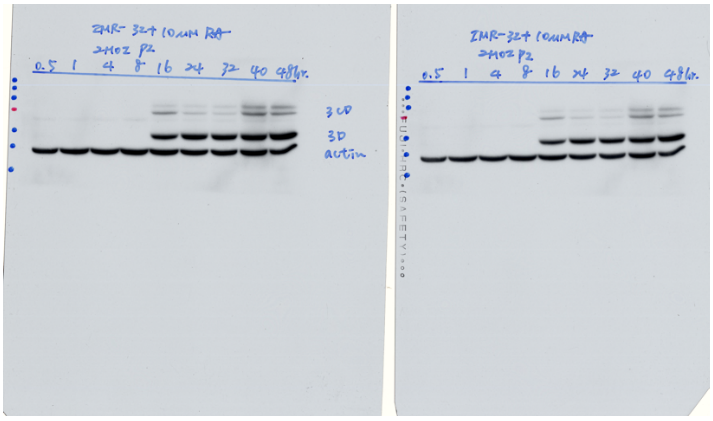

Figure 4G

Phospho-Beclin1 S15

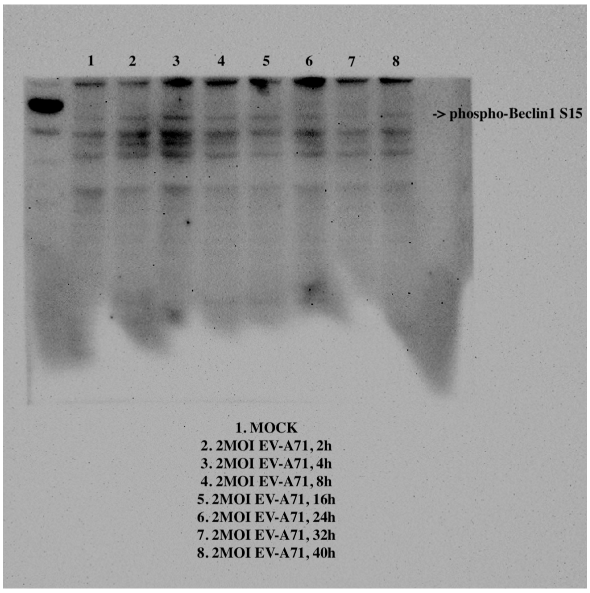

Total Beclin1

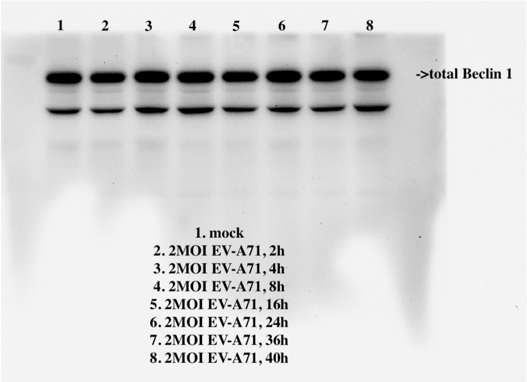

EV-A71 3D

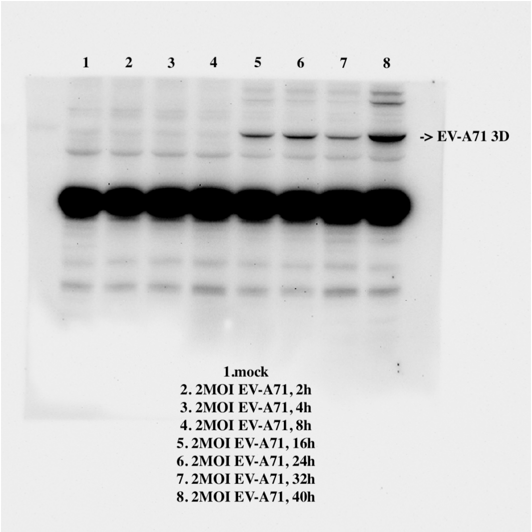

actin

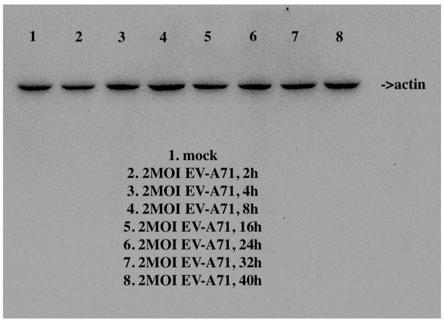

Figure 5A

p62

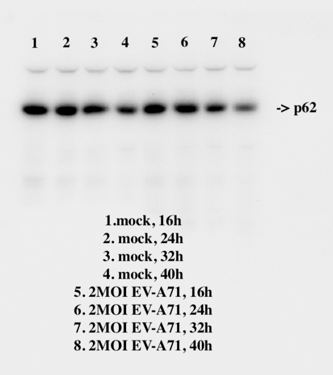

EV-A71 3D

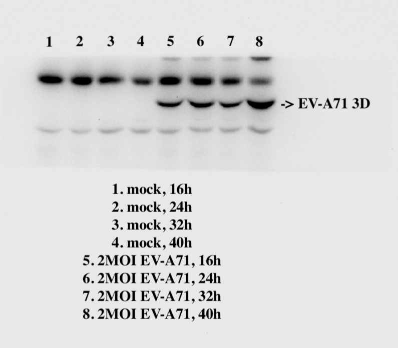

actin

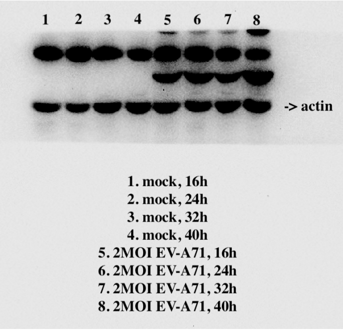

Figure 5E

LC3 I/ LC3II

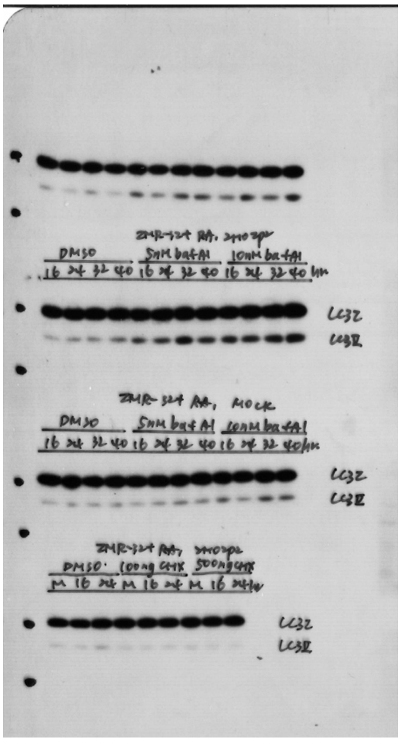

EV-A71 3D

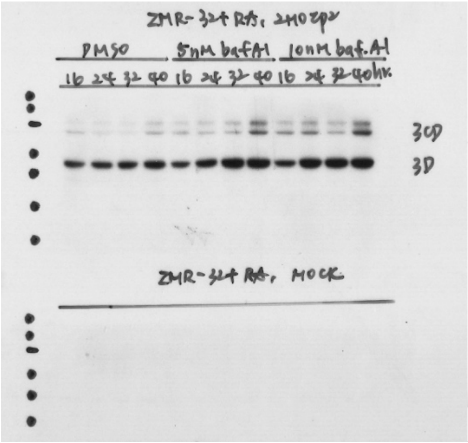

actin

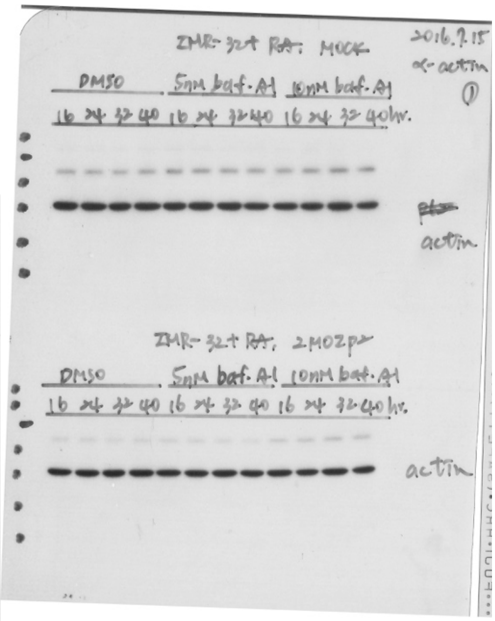

Figure 5G

LC3 I/ LC3II

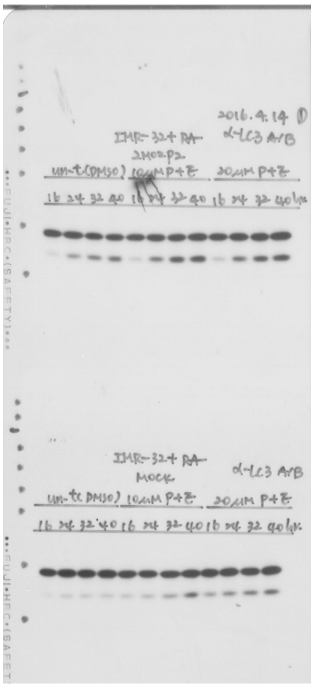

EV-A71 3D

actin

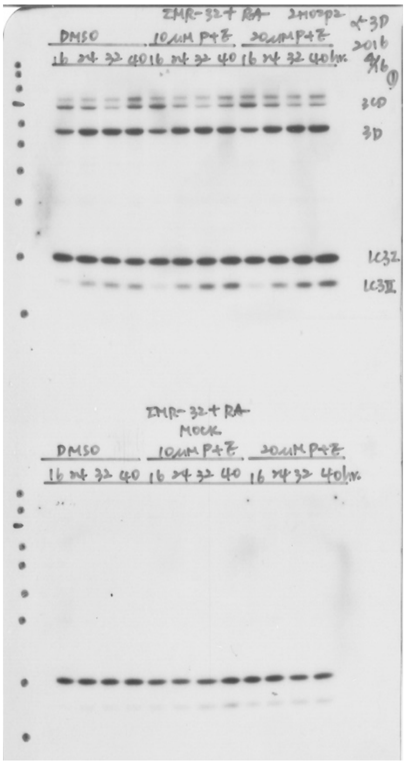

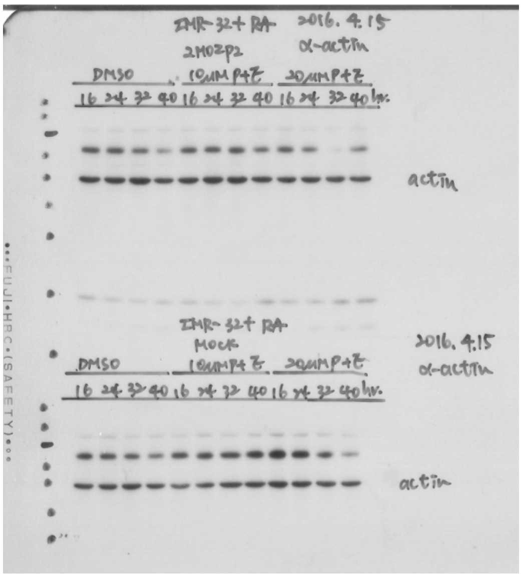

Figure 6A

LC3I/ LC3II

EV-A71 3D

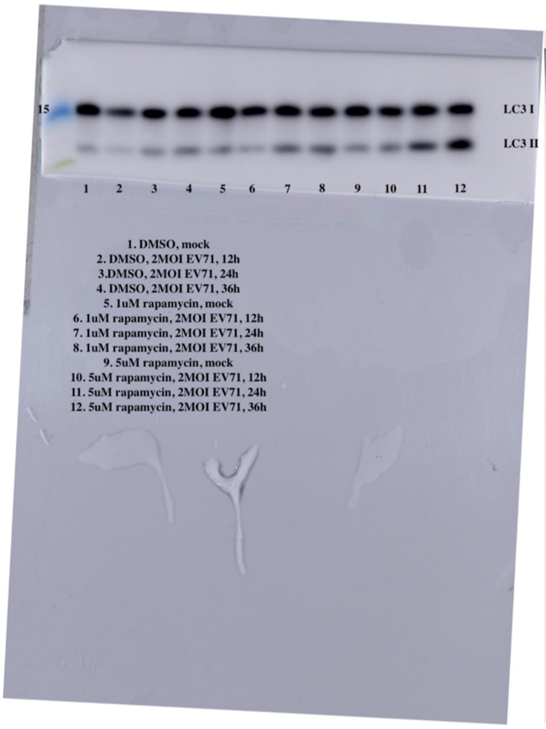

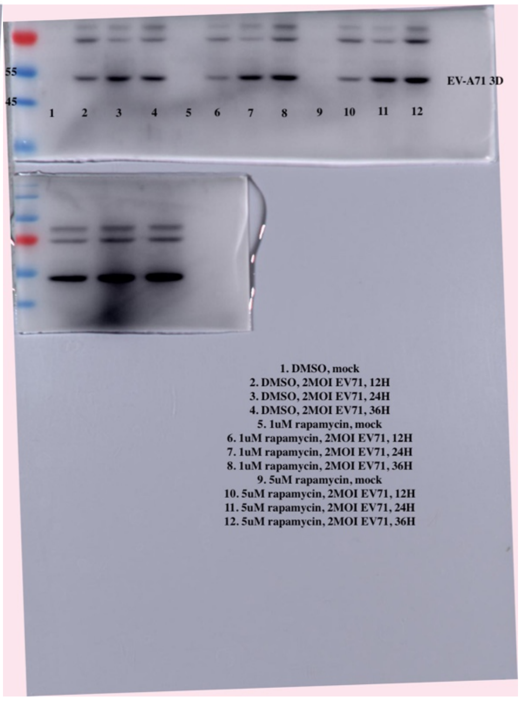

actin

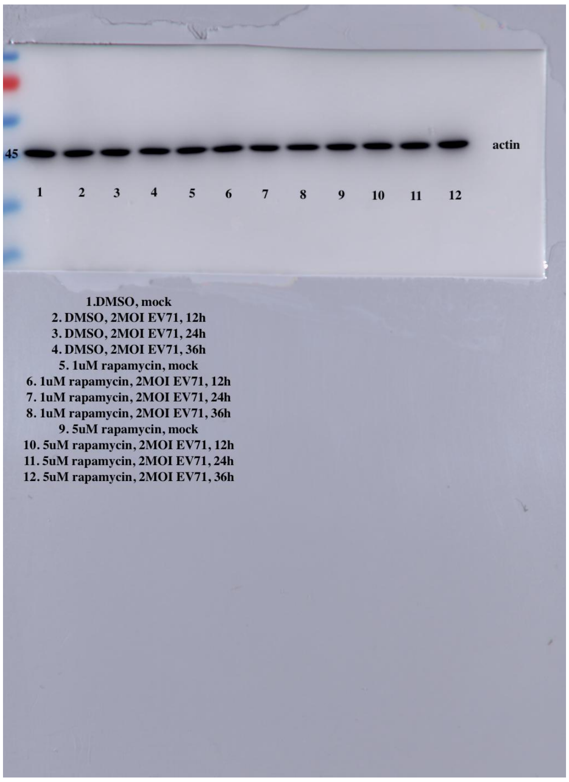

Figure 6C

LC3I/ LC3II

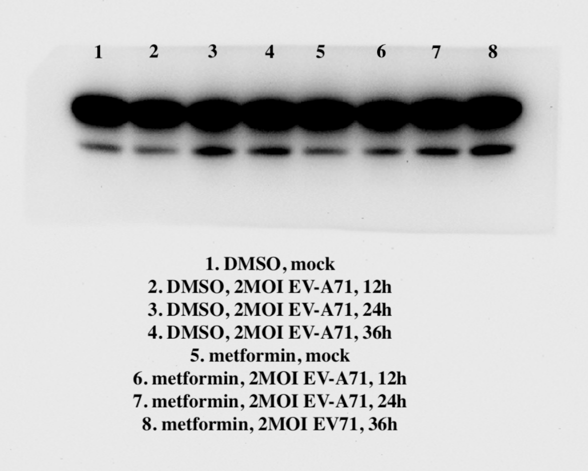

EV-A71 3D

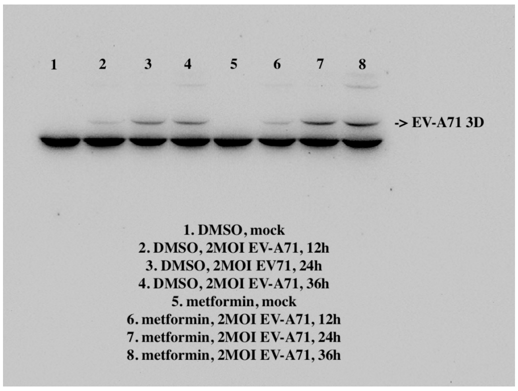

actin

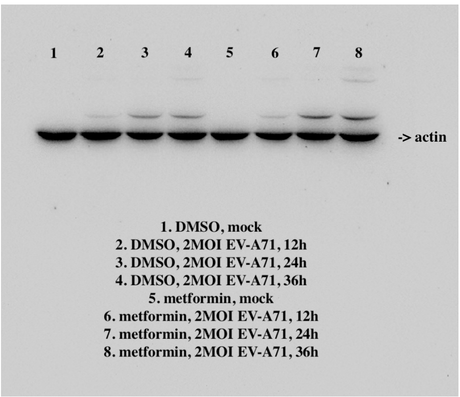

Figure 6F

ATG5

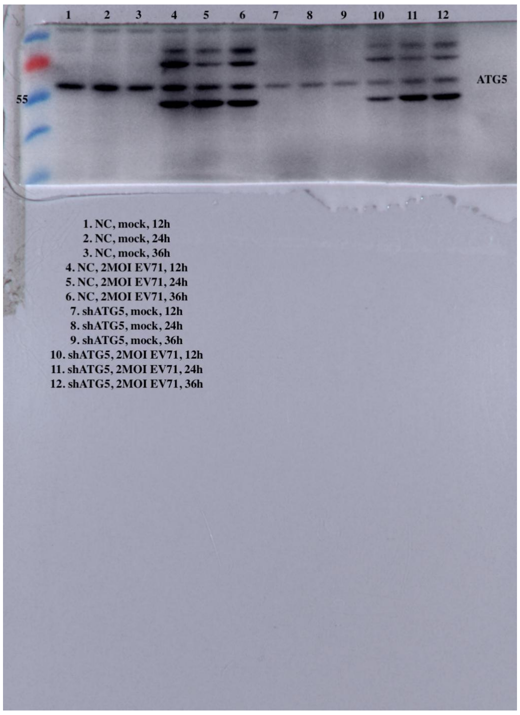

LC3I/ LC3 II

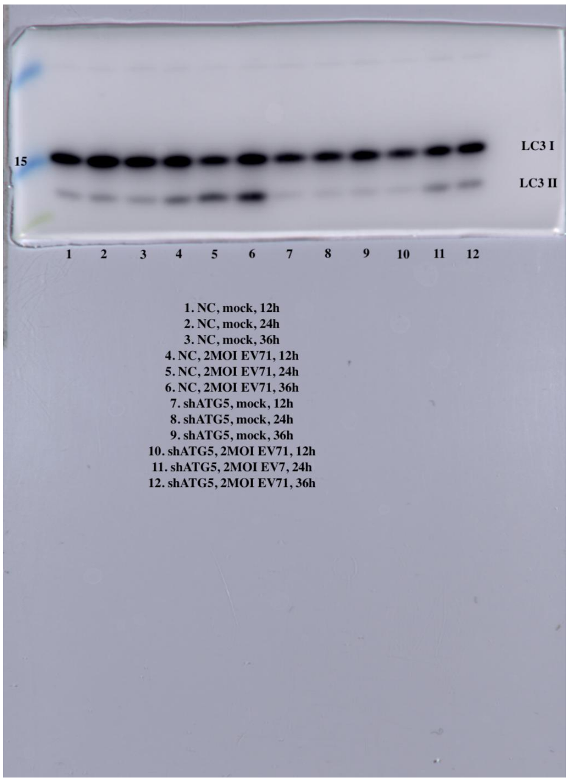

EV-A71 3D

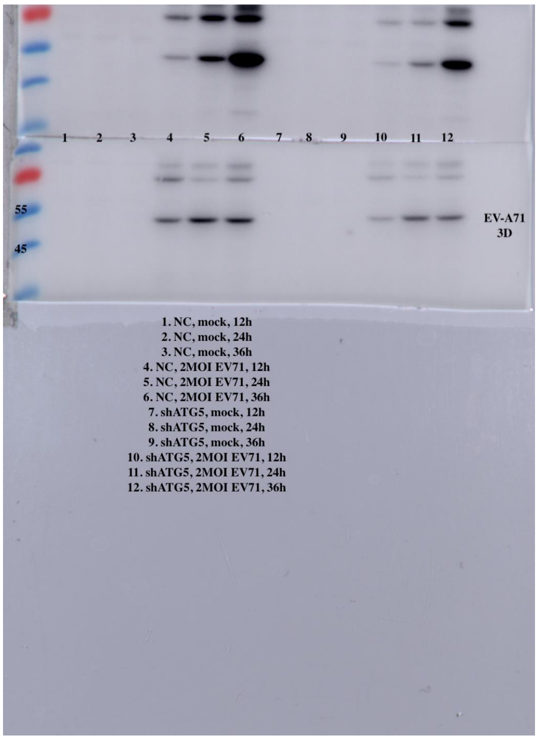

actin

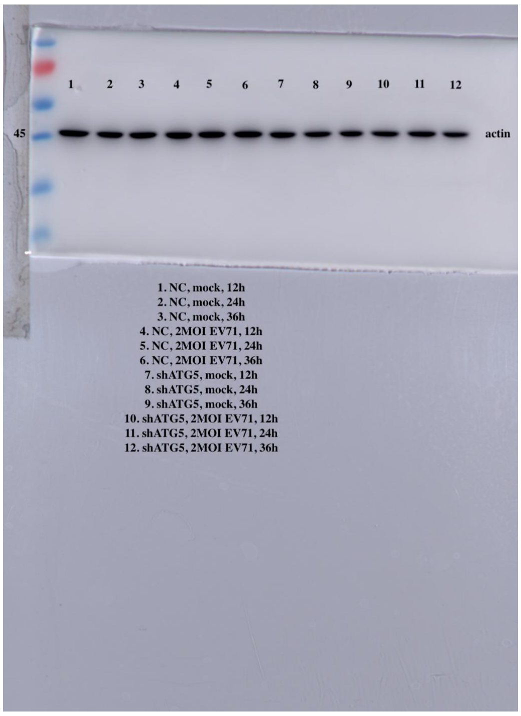

Figure S3A

LC3-I/LC3-II

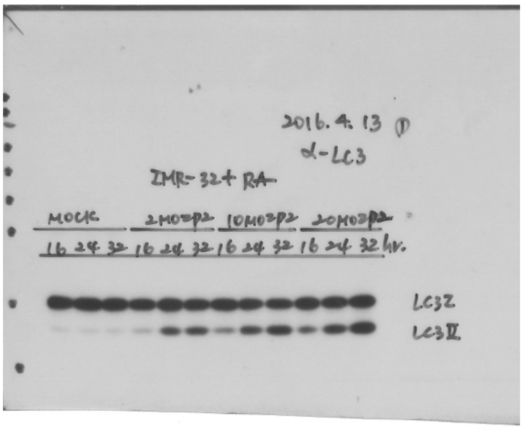

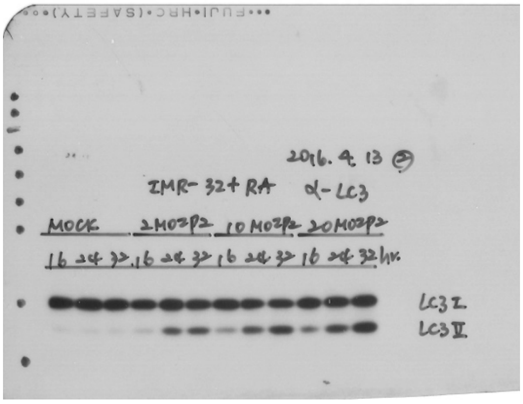

EV-A71 3D

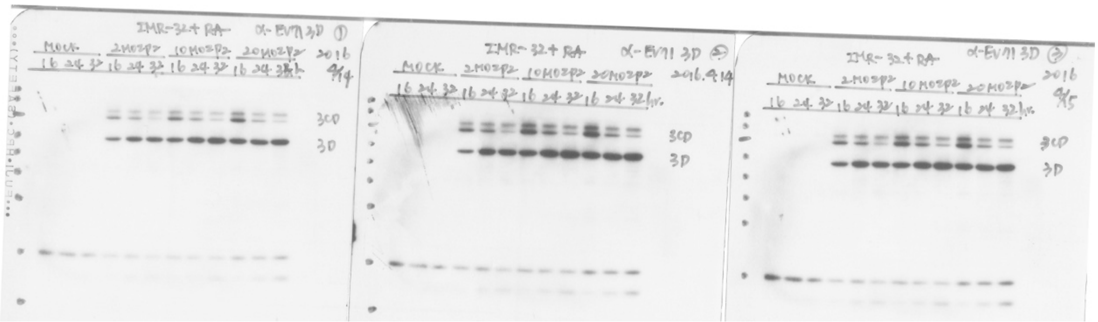

actin

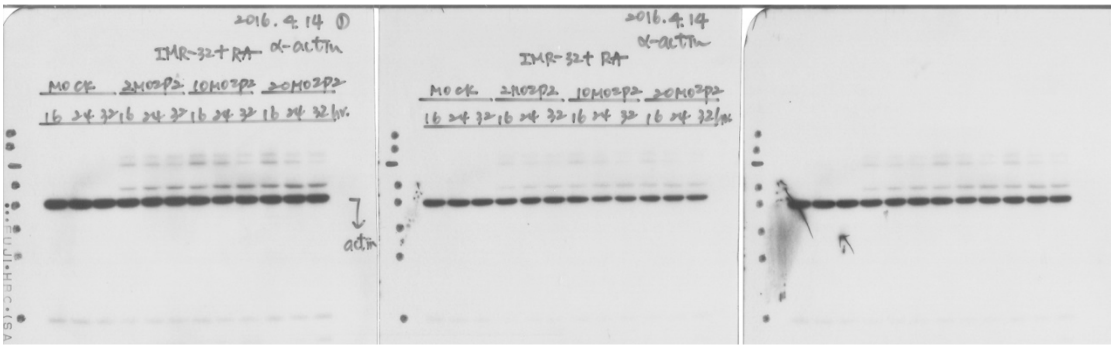

Figure S3B

EV-A71 3D/ actin

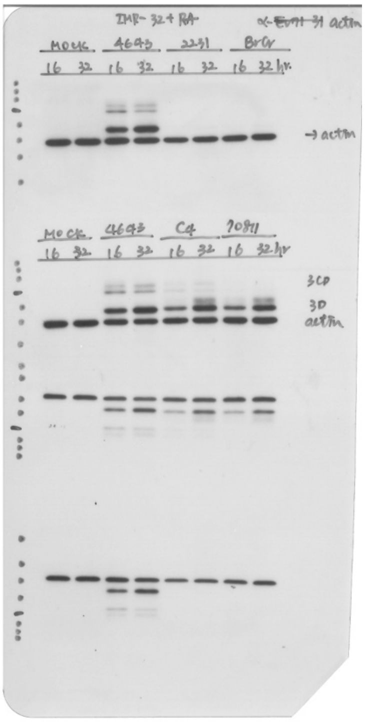

LC3 I/ LC3 II

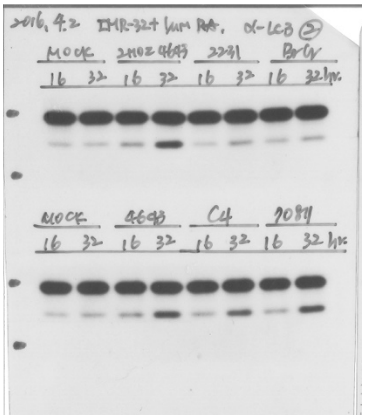

Supplement: Supplementary file 2 — Supplementary Information 2. [file 41598_2020_71970_MOESM2_ESM.pdf]
